# Plasma short-chain fatty acid concentrations in social anxiety disorder and changes after cognitive behavioral therapy

**DOI:** 10.1038/s41398-026-04134-y

**Published:** 2026-06-03

**Authors:** Wenjie Cai, Miranda Stiernborg, Alexander Wolthon, Rikard Landberg, Amirhossein Manzouri, Tomas Furmark, Catharina Lavebratt, Kristoffer N. T. Månsson

**Affiliations:** 1 Department of Molecular Medicine and Surgery, Karolinska Institutet, Stockholm, Sweden; 2 Karolinska University Hospital Solna, Center for Molecular Medicine, Stockholm, Sweden; 3 Department of Psychology, Stockholm University, Stockholm, Sweden; 4 Department of Comparative Medicine, Karolinska Institutet, Stockholm, Sweden; 5 Department of Life Sciences, Division of Food and Nutrition Science, Chalmers University of Technology, Gothenburg, Sweden; 6 Department of Clinical Neuroscience, Karolinska Institutet, Stockholm, Sweden; 7 Centre for Psychiatry Research, Department of Clinical Neuroscience, Karolinska Institutet, & Stockholm Health Care Services, Region Stockholm, Stockholm, Sweden; 8 Department of Clinical Psychology and Psychotherapy, Babeș-Bolyai University, Cluj-Napoca, Romania; 9 Department of Psychology, Uppsala University, Uppsala, Sweden

**Keywords:** Psychology, Psychiatric disorders

## Abstract

Emerging evidence indicates that the gut microbiota are linked to variation in social behavior and anxiety, with short-chain fatty acids (SCFAs) proposed as key microbial metabolites that may mediate microbiota-brain communication. Cognitive behavioral therapy (CBT) is an effective treatment for social anxiety disorder (SAD), but it remains unclear whether gut microbiota changes following treatment. In this longitudinal study, 46 individuals with SAD underwent nine weeks of internet-delivered CBT. Plasma concentrations of nine SCFAs were measured using liquid chromatography-mass spectrometry: twice at pre-treatment, once at post-treatment and once at a 36-month follow-up in patients, with > 80% data completeness. Healthy controls (*n* = 42) underwent two SCFA assessments 11 weeks apart. Generalized additive mixed models were used to assess longitudinal changes with CBT, linear mixed models for patient-control differences, and intraclass correlation coefficients for test-retest reliability. CBT led to significant reductions in social anxiety symptoms, and effects were sustained at 36 months follow-up. Plasma concentrations of butyric, isobutyric, propionic, and valeric acids increased after CBT, with trajectories predominantly explained by time rather than individual variability. Before CBT, isobutyric acid was statistically significantly lower in SAD patients, relative to healthy controls. These findings provide initial evidence that sustained therapeutic benefits of CBT may be linked to alterations in circulating microbial metabolites, as indexed by lower isobutyric acid in SAD patients at pre-treatment, and increases in isobutyric, butyric, propionic and valeric acids after therapy. Lifestyle factors were not assessed but may have contributed to the observed long-term metabolic changes.

## Introduction

Social anxiety disorder (SAD) is a debilitating psychiatric disorder characterized by an excessive fear of social situations and negative evaluation by others, with a lifetime prevalence exceeding 12% [1]. SAD severely impairs individuals’ social functioning, manifesting as decreased work productivity, increased school dropout, elevated unemployment rates, and impaired social and romantic relationships [2, 3]. The disorder is also associated with substance abuse, severe depression, and an increased risk of suicide [3, 4]. Twin studies have shown that genetic factors play an important role [5] as well as interactions with environmental factors, including parenting styles and adverse life events [6].

Animal studies have consistently shown that an altered microbiota-gut-brain axis state is associated with social behavior. Recent reports suggest that patients with SAD exhibit an altered microbial composition compared with healthy controls [7] and that germ-free mice receiving faecal transplants from SAD patients exhibit high sensitivity to social fear [8]. Thus, an altered microbiota-gut-brain axis state may contribute to the pathophysiology of SAD. Short-chain fatty acids (SCFAs) are produced by the bacterial fermentation of dietary fibre in the gut and are considered essential mediators of microbiota-gut-brain communication. SCFAs play vital roles not only in maintaining gastrointestinal health but also in systemic metabolism [9], immune regulation [10, 11], and critical neurological processes, such as synaptic plasticity, neural differentiation, and neurotransmitter regulation [12, 13]. Emerging evidence links altered SCFA profiles with various mental disorders, including ADHD [14], autism [15] and depression [16]. A recent experimental study shows that acute stress can rapidly reduce SCFAs availability and disrupt gut-brain barrier function [17], highlighting SCFAs as dynamic, stress-sensitive microbial metabolites with direct relevance for psychopathology. Understanding how these SCFA patterns influence treatment outcomes is therefore a key research topic.

Cognitive behavioral therapy (CBT) has emerged as a partly effective treatment for SAD, with internet-delivered guided self-help programs being an accessible and efficacious alternative [18, 19]. These programs involve structured modules with homework assignments and therapist guidance, facilitating treatment adherence and consistency across participants [20, 21]. The therapeutic gains are well maintained over the long term [22]. Despite the effectiveness of CBT, many patients’ outcomes remain unsatisfactory. One approach to better understand how effective treatment works is to investigate the biological mechanisms underlying the therapeutic response. To this end, we have previously demonstrated that CBT is associated with structural and functional neural plasticity, including reduced amygdala volume [23] and that pre-treatment neural variability reliably predicts CBT outcome [24, 25]. Further, we have previously reported that improvements in cellular protective markers (i.e., glutathione peroxidase and telomerase activity) increased after CBT [26], and that circulating cell-free mitochondrial DNA in peripheral blood was reduced in SAD patients relative to matched controls [27].

To our knowledge, there are currently no data on plasma concentrations of SCFA in patients with SAD, and no longitudinal studies investigating changes in plasma SCFA profiles after effective treatment. Our study aimed to (1) examine the short- and long-term changes in SCFAs concentrations and treatment outcomes following internet-delivered CBT in SAD patients, and (2) to compare pre-treatment SCFA concentrations in SAD patients relative to matched healthy controls.

## Materials and methods

### Participant and study design

The recruitment procedures have been described in detail, and some clinical outcomes have been reported elsewhere [24, 26, 28]. In brief, participants aged over 18 were recruited through media advertising as previously described. The Mini-International Neuropsychiatric Interview (M.I.N.I; v7) and the Structured Clinical Interview for DSM-IV-Axis I Disorders (SCID-I) were used for the diagnosis of SAD [29, 30]. Somatic and neurological conditions were screened via self-report at baseline, and structural magnetic resonance imaging scans were reviewed by a neuroradiologist to exclude clinically relevant abnormalities. Individuals with neurological diseases, bipolar disorder, psychotic disorders, severe depression, alcohol or substance abuse disorders, or antisocial personalities were excluded. Ultimately, 46 patients diagnosed with SAD were enrolled in the study (Table 1). None of the participants were undergoing psychotherapy concurrently during the study’s active treatment period, and four participants (9%) were on psychoactive drug treatment and agreed to maintain a stable medication dosage for at least three months before and during CBT. In addition to the SAD patients, a matched healthy control (HC) group was also recruited (Table 1), comprising individuals with no life-time history or current diagnosis of psychiatric disorders, as assessed by M.I.N.I. (modified to assess life-time history). There were no significant differences between SAD patients and HC participants regarding sex, age, BMI, or smoking status (all *P’s* > 0.20). Screening and enrollment of SAD participants and healthy controls were conducted on separate occasions. For clarity, Supplementary Fig. S1-2 presents the timing of all assessments in relative weeks. The Insomnia Severity Index (ISI) [31] is a self-administered questionnaire used to assess insomnia symptoms.

**Table 1:** Demographic characteristics.

	SAD patients (*n* = 46)	Healthy controls (*n* = 42)
**Sex,** ***n*** **(%)**		
Females	29 (63.0)	24.0 (57.1)
Male	17 (37.0)	18.0 (42.9)
**Age, mean (SD)**	30.7 (8.3)	31.9 (9.5)
**BMI, mean (SD)**	24.8 (4.4)	23.8 (3.2)
**SAD, duration in years, mean (SD)**	17.0 (9.9)	n/a
**Smoking regularly the past 3 months,** ***n*** **(%)**	4 (8.7)	8 (19.0)
**Marital status,** ***n*** **(%)**		
Married/cohabiting with children	16 (34.8)	15 (35.7)
Married/cohabiting without children	10 (21.7)	12 (28.6)
Non-cohabiting partner	4 (8.7)	3 (7.1)
Single with children	4 (8.7)	2 (4.8)
Single without children	9 (19.6)	9 (21.4)
Other	3 (6.5)	1 (2.4)
**Education,** ***n*** **(%)**		
Completed primary school	3 (6.5)	0 (0.0)
Completed secondary school	7 (15.2)	5 (11.9)
Completed vocational education	2 (4.3)	2 (4.8)
Ongoing university education	16 (34.8)	13 (31.0)
Completed university education	18 (39.1)	22 (52.4)
**Concurrent medications,** ***n*** **(%)**		
No medication	18 (39.1)	n/a
SSRIs	4 (8.7)	n/a
Hormonal contraceptives	16 (34.8)	11 (26.2)
Antihistamines	1 (2.2)	n/a
Hormone medications	1 (2.2)	n/a
Thyroid hormone substitution	2 (4.4)	n/a
**Concurrent comorbidity,** ***n*** **(%)**		
No concurrent psychiatric comorbidity	12 (26.1)	n/a
Major depressive disorder	3 (6.5)	n/a
Panic disorder	3 (6.5)	n/a
Agoraphobia	5 (10.9)	n/a
Generalized anxiety disorder	3 (6.5)	n/a
Binge eating disorder	1 (2.2)	n/a
Obsessive compulsive disorder	2 (4.3)	n/a

**Fig. 1:**
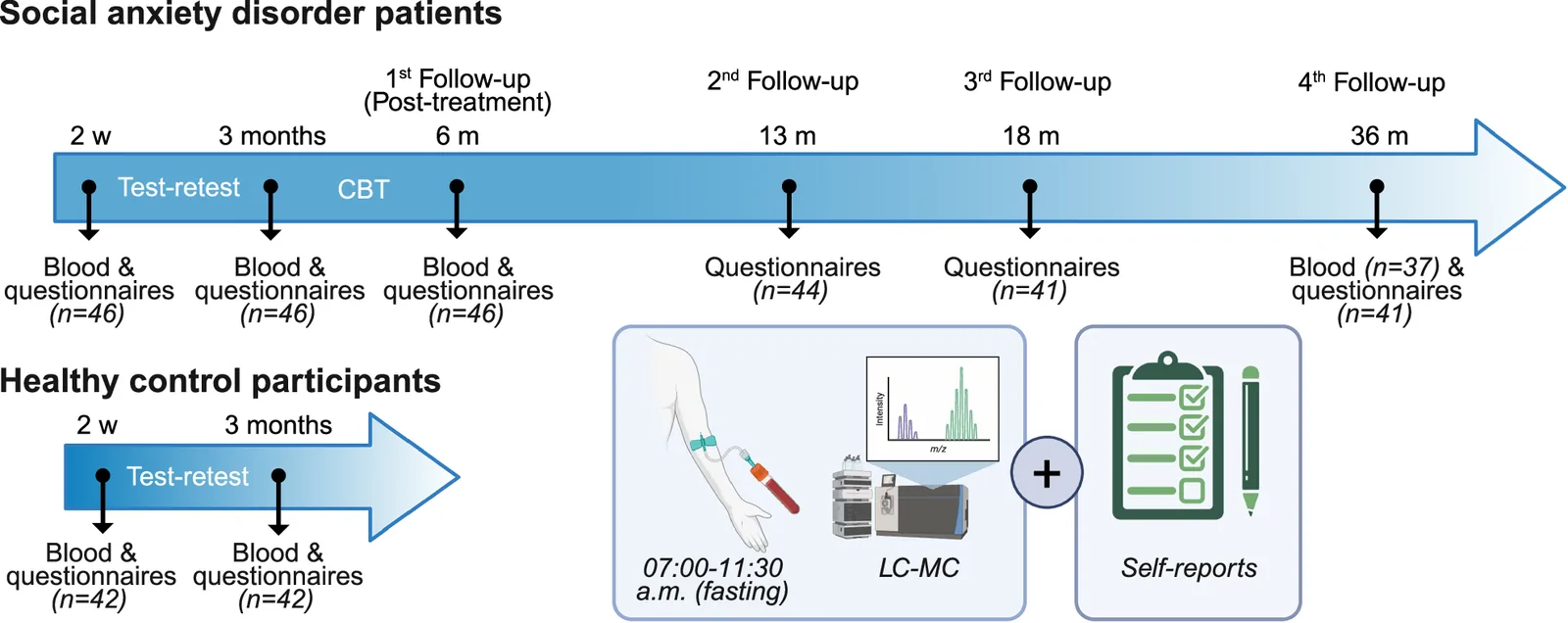
Study design and timeline of assessments for patients with social anxiety disorder and matched healthy controls. All participants underwent two baseline assessments, including clinical self-reports and blood draws. Patients completed four additional assessments after receiving cognitive behavioural therapy: (1) follow-up assessment at post-treatment (~6 months after recruitment, including blood draw); (2) assessment at ~13 months (self-reports only); (3) assessment at ~18 months (self-reports only), and (4) final assessment at ~36 months including both self-reports and blood draw. Created with BioRender.com.

As shown in Fig. 1, all participants completed two baseline assessments, and SAD patients underwent four additional follow-ups after CBT (post-treatment, and at ~13, ~18, and ~36 months). Exact numbers of biological and self-report assessments across time-points are shown in Fig. 1.

### Internet-delivered cognitive behavioral therapy for social anxiety disorder

Internet-delivered CBT is a self-guided psychological treatment intervention based on principles of traditional CBT [19]. Patients received one module per week over nine weeks, with each module including text materials and homework assignments. A clinical psychologist interacted weekly with participants through messages to provide guidance and feedback, see also [32] for details.

### Clinical assessments

The Liebowitz Social Anxiety Scale Self-Report version (LSAS-SR) was used as the primary outcome measure. LSAS-SR is a widely used and validated psychological instrument specifically designed for assessing the severity of SAD [33–35]. In the Supplementary Table S1-3, data on the Montgomery-Åsberg Depression Rating Scale – Self-rated version (MADRS-S) scores are reported.

### Analysis of plasma short-chain fatty acids

Participants were instructed to begin fasting at 10:00 p.m. the evening before blood collection, and all blood samples were collected by an experienced research nurse the following morning between 7:00 and 11:30 a.m. Plasma was isolated from lithium-heparin tubes immediately after sampling and stored at −80 °C. Without prior freeze-thawing, the plasma was thawed, and plasma concentrations of nine SCFAs (i.e., formic, acetic, propionic, butyric, isobutyric, succinic, valeric, isovaleric, and caproic acids) were analyzed using liquid chromatography-mass spectrometry (LC-MS) [36] at the Department of Life Sciences, Chalmers University of Technology, Gothenburg, October 2021. Per analysis batch, 40 samples from SAD and controls were analyzed in singlets, together with 3 quality control samples (QC) in triplicates. All samples from a given individual were processed in the same batch, ensuring even distribution of timepoints within batches. In total, 6 batches were run. The QC samples had SCFAs concentrations within the range of the human samples and were used to calculate the within-batch coefficient of variation (CV) of 7.4%, 3.4%, 9.5%, 8.7%, 8.6%, 11.4%, 17.3%, 7.4%, and 8.2% for formic, acetic, propionic, butyric, isobutyric, succinic, valeric, isovaleric, and caproic acid, respectively. Between-batch variation was controlled by normalization of the individual data across batches, where each value was divided by the normalization factor (mean of the QC values of the individual batch)/(mean of the QC values from all batches). SCFA concentrations have not been reported in previous publications on this cohort.

### Statistical analysis

All analyses were conducted in R (v4.5.0; R Core Team, 2022) via RStudio (v.2025.5.0.496, Posit, Boston, MA, USA), and all statistical tests were two-tailed with an alpha level set at 0.05. The primary analysis was a longitudinal within-patient analysis across repeated assessments. For reference, 46 patients provide 91% power to detect a moderate pre–post effect (Cohen’s d = 0.5) at α = 0.05. To examine short- and long-term changes in clinical outcomes and SCFA concentrations, generalized additive mixed models (GAMMs) were used. Non-linear trajectories over time were represented by penalized regression splines, and individual variability was accounted for via random effects. For clinical data, which included repeated measurements at seven time points, both random intercepts and random slopes for time were included. For SCFA outcomes, which were sampled at four time points per patient, random intercepts were used exclusively, as models with random slopes frequently resulted in singular fits or unstable variance estimates. Models were estimated using the mgcv package [37], v.1.9.1), and summaries included effective degrees of freedom (edf), F-statistics, *P* values, and deviance explained. Marginal and conditional *R²* values were estimated according to the Nakagawa method [38], using the performance package [39], v.0.13.0) based on refitted models using the gamm4 package (https://CRAN.R-project.org/package=gamm4, v.0.2.7). Paired Cohen’s *d* effect sizes were computed to quantify the magnitude of change in LSAS-SR scores. Specifically, effect sizes were calculated for paired comparisons between the second baseline and both the first and the last post-treatment follow-ups. The statistical analysis of SCFA concentrations was conducted using normalized values. SCFA values were missing for four HCs at the first baseline, and for one HC at the second baseline. Three participants, with samples identified as extreme outliers exceeding 8 times the interquartile range (IQR), were removed from all analyses (see Supplementary Fig. S3 for details).

Group comparisons of LSAS-SR scores and SCFA concentrations between the SAD patients and HC participants were examined between the two baseline time points, using linear mixed-effects models (LMM) implemented in the lme4 package ([40], v.1.1.37), estimating fixed effects of group, time, and their interaction. Random intercepts were included for each participant to account for within-subject dependencies. The models used sum-coded contrasts to reflect the structure of a 2 × 2 repeated-measures ANOVA, with main effects representing marginal means across levels of the other factor. Corresponding Cohen’s *d* effect sizes were computed for group and time comparisons. *P* values for SCFA outcomes in the longitudinal (GAMM) and baseline group comparison (LMM) analyses were adjusted for multiple comparisons using the Benjamini-Hochberg method to control the false discovery rate. Adjusted *P* values (*P*_FDR_) below 0.05 were considered statistically significant.

To assess the test-retest reliability between the two baseline assessments in both SAD patients and HC participants, single-measurement, absolute-agreement, two-way mixed-effects intraclass correlation coefficients (ICC) were computed by using the psych package ([41], v2.5.6), and interpreted based on the guidelines by McGraw & Wong [42].

Exploratory analyses included within-group paired comparisons of ISI across time (pre- to post-treatment; and post-treatment to 36-month follow-up) using paired-samples t-tests, with effect sizes quantified using Cohen’s *d*. Associations between SCFA concentrations and LSAS-SR were examined using Pearson correlations at each baseline time point. Further, we also investigated whether pre-treatment SCFA concentrations were predictive of subsequent changes in LSAS-SR (pre- to post-treatment; and post-treatment to the 36-month follow-up). In addition, Pearson correlation tests were used to examine associations between changes in SCFA concentrations and changes in ISI scores over the corresponding intervals (baseline to post-treatment; post-treatment to the 36-month follow-up). Assumptions for Pearson-based inference were evaluated using residual diagnostics from corresponding linear models (outcome ~ predictor), including Shapiro-Wilk tests of model residuals and inspection of QQ-plots.

## Results

### Social anxiety severity changes after cognitive behavioral therapy

**Fig. 2:**
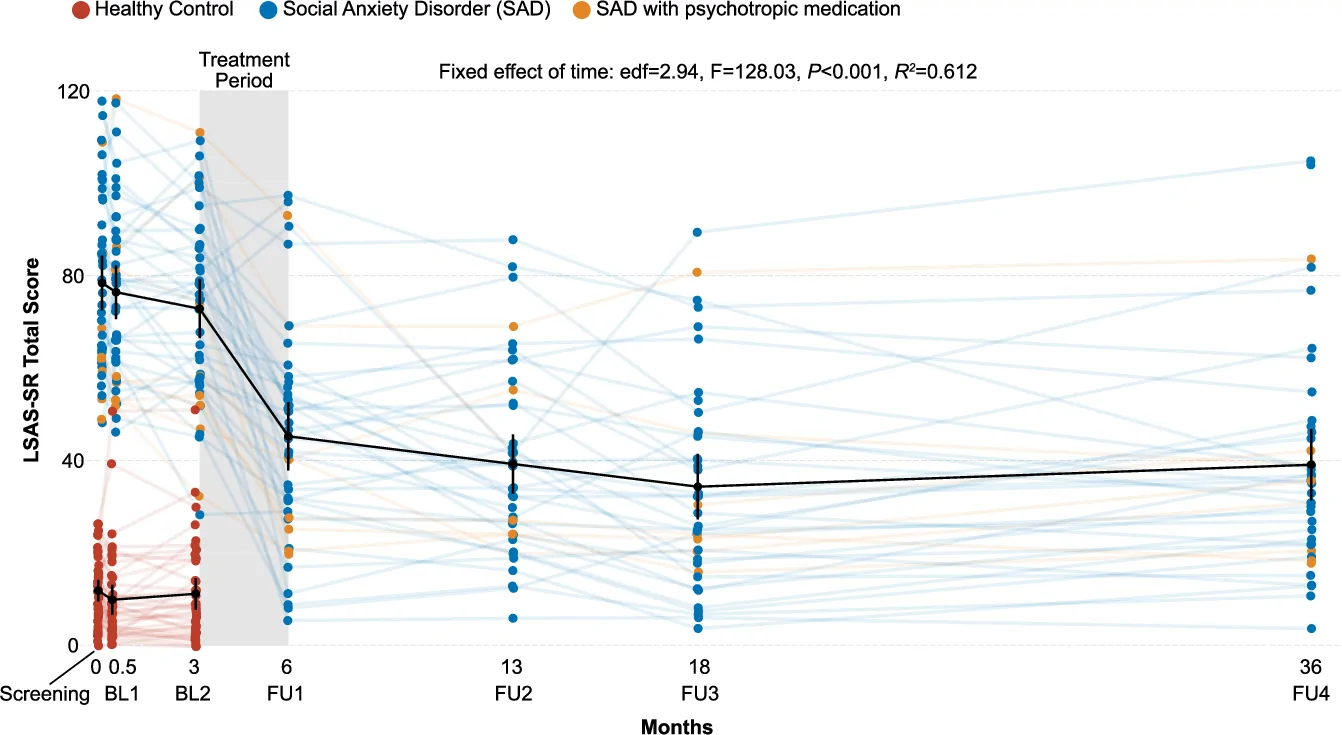
Trajectories of social anxiety symptoms over time estimated using generalized additive mixed models. Total scores on the Liebowitz Social Anxiety Scale Self-Report version (LSAS-SR), for individuals with social anxiety disorder (SAD), and healthy controls are presented. Four SAD patients with concurrent psychotropic medication are marked in orange color. Each dot represents an individual measurement, and lines connect repeated measures for each participant. The grey shaded area marks the 9-week internet-delivered cognitive behavioral therapy (CBT) treatment period. Error bars represent 95% CI. BL1 1st baseline assessment, BL2 2nd baseline assessment, FU1 1st follow-up assessment, FU2 2nd follow-up assessment, FU3 3rd follow-up assessment, FU4 4th follow-up assessment.

LSAS-SR scores decreased non-linearly over time following CBT, with the largest reduction during the treatment period, followed by smaller continued reductions over the subsequent months (Fig. 2). The fixed effect of time was significant and explained most of the variance (*P* < 0.001, *R*^*2*^ = 0.61, relative to the random effect *P* < 0.001, *R*^*2*^ = 0.23). The paired Cohen’s *d* representing the change from the second baseline to the post-treatment was 1.22 (95% CI: 0.81–1.64), and 1.51 (95% CI: 0.97–2.05) for the change from the second baseline to the 36-month follow-up.

### Short-chain fatty acid changes during and after cognitive behavioral therapy

**Fig. 3:**
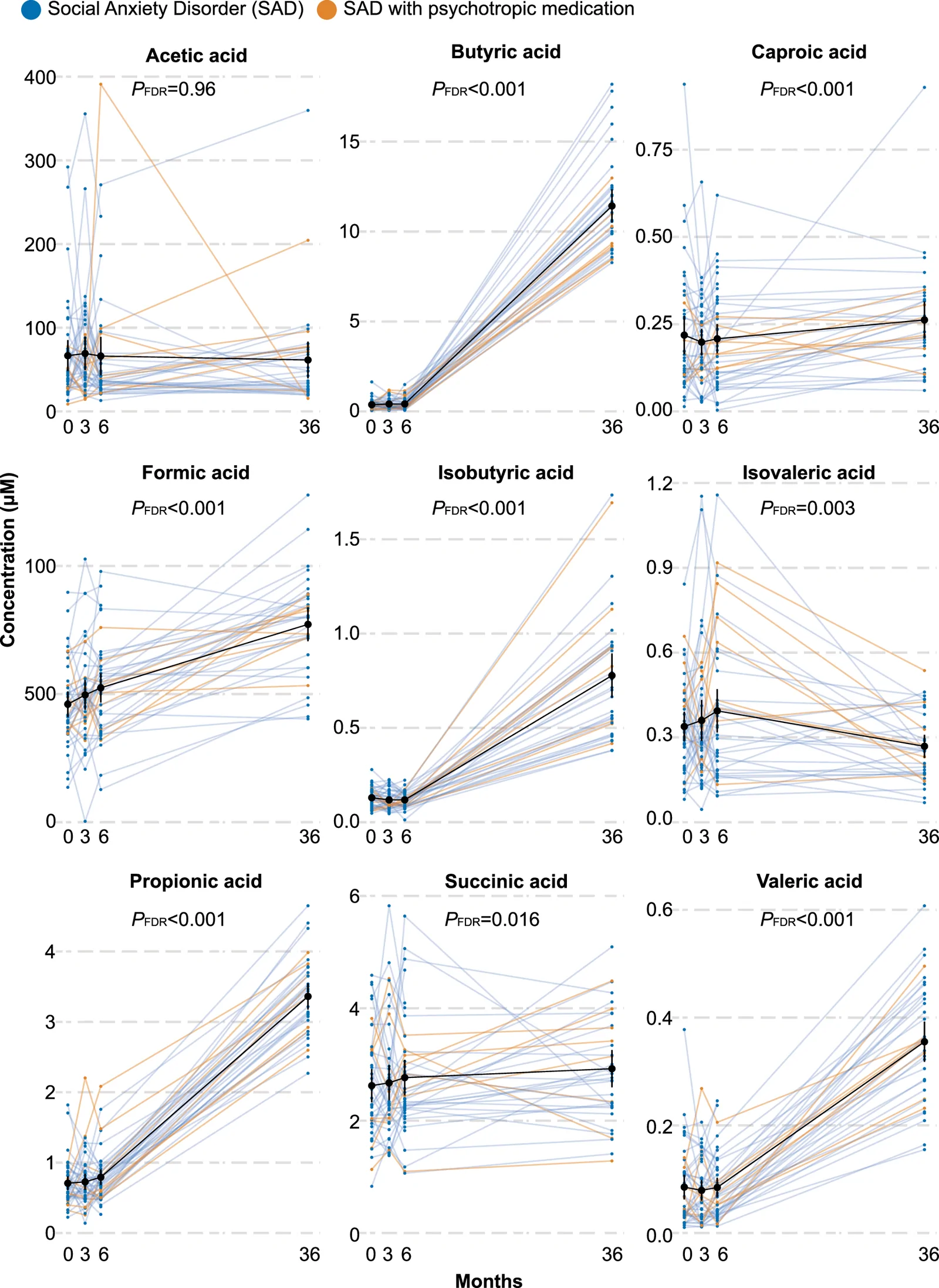
Short-chain fatty acids change in patients with social anxiety disorder (SAD) across the weeks. The concentration of each SCFA (μM) in SAD patients over four time points: two baseline assessments (1st baseline assessment at 2 weeks and 2nd baseline assessment at 3 months after recruitment), first follow-up assessments at post-treatment (~6 months) and the final assessment at 39 months after recruitment. Orange lines represent individual SAD patients with concurrent psychotropic medication (*n* = 4). Outliers are excluded, and solid black error bars represent 95% CI. FDR corrected *P (*estimated using generalized additive mixed models), based on the effect of time are displayed.

As presented in Fig. 3 and Table S1, butyric, isobutyric, propionic, and valeric acid increased non-linearly over time, with limited change between baseline and post-treatment, and more pronounced increases between post-treatment and the 36-month follow-up. Most of the variance was explained by time (*P*_FDR_ < 0.001, *R²* = 0.60–0.89), rather than individual variability (*P*_FDR_ = 0.001–0.47, *R*^*2*^ = 0.001–0.22). Isovaleric acid changed non-linearly over time as well, but with a different trajectory, peaking at post-treatment and declining below baseline levels at the 36-month follow-up (with individual variability accounting for most variance, *P*_FDR_ < 0.001, *R*^*2*^ = 0.43, instead of time (*P*_FDR_ = 0.003, *R²* < 0.001).

Caproic, formic, and succinic increased more linearly over time. Of these, formic acid showed the greatest change and was largely explained by time (*P*_FDR_ < 0.001, *R*^*2*^ = 0.54), while the relatively modest changes in caproic and succinic acid were only to a smaller extent explained by time (*P*_FDR_ 0.001–0.016, *R*^*2*^ = 0.029–0.038). Acetic acid did not change over time (*P*_FDR_ = 0.96, *R²* < 0.001), and individual variability was statistically significant for this measure (*P*_FDR_ < 0.001). For model statistics and acid trajectories over time, see Supplementary Table S1 and Fig. 3, respectively.

### Differences between patients and healthy controls

**Fig. 4:**
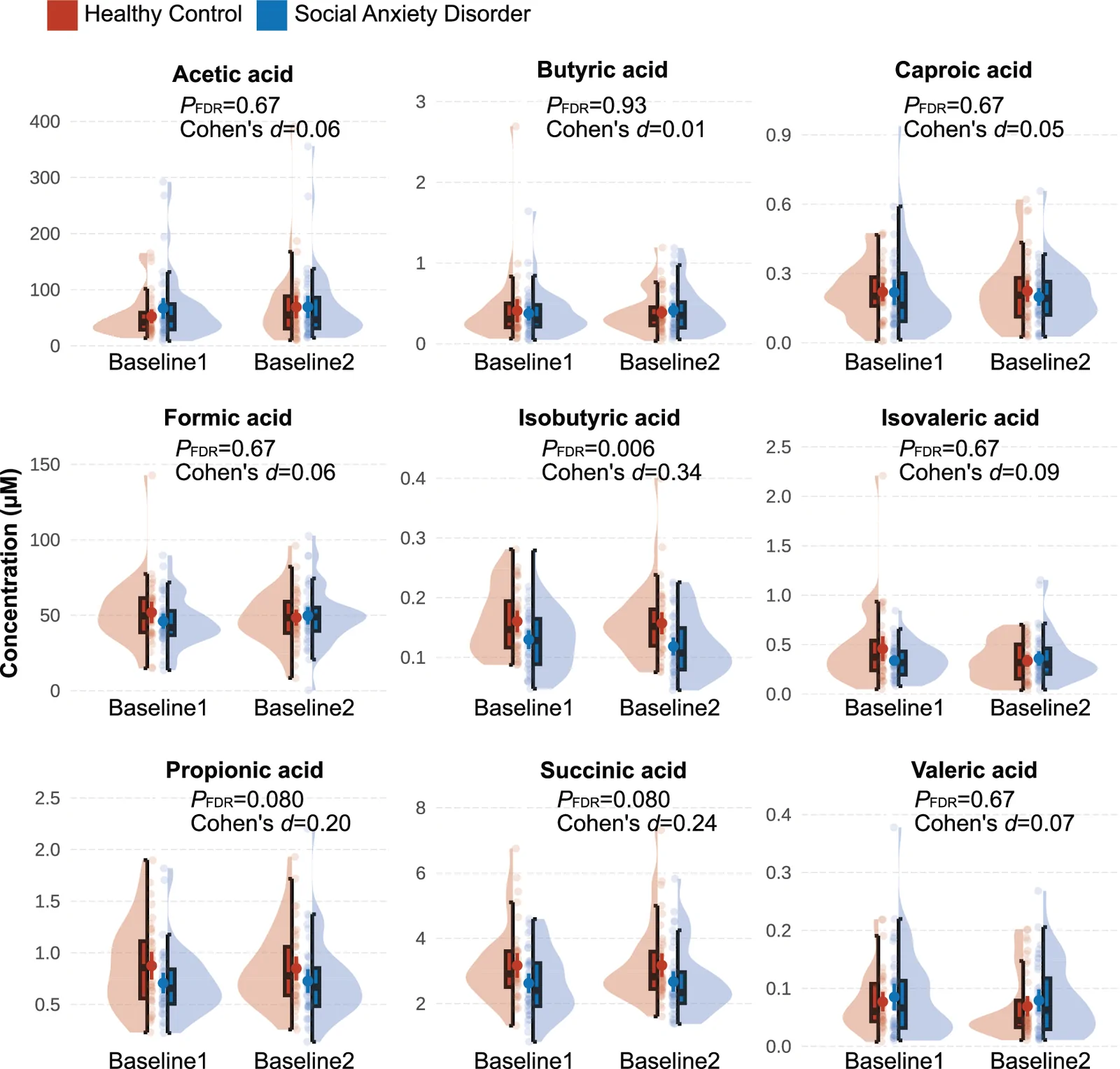
Short-chain acid concentration differences in plasma between patients with social anxiety disorder (SAD) and healthy controls (HCs) at baseline. The concentration (μM) of each SCFA in SAD patients (blue) and HCs (red) at the first baseline. The black dot represents the mean, and colored solid error bars represent 95% CI.

LSAS-SR scores were significantly higher in SAD (M ± SD = 74.63 ± 19.81) patients than in HC (M ± SD = 10.49 ± 10.47) participants at baseline (*P* < 0.001, *d* = 2.01; see Fig. 2). At both baselines, isobutyric concentration in SAD patients (M ± SD = 0.12 ± 0.05) was lower relative to HCs (M ± SD = 0.16 ± 0.06; *P*_FDR_ = 0.006, Cohen’s *d* = 0.34). No other SCFA showed FDR corrected statistically significant differences between SAD patients and HCs (*P*_FDR_ ≥ 0.08; Fig. 4, Supplementary Table S2).

### Test-retest reliability of clinical assessments and short-chain fatty acid concentrations

Test-retest reliability between the two baselines (spanning 2.5 months) was good to excellent for the LSAS-SR for both SAD (ICC = 0.89) and HCs (ICC = 0.83). SCFA outcomes demonstrated higher variability, with ICCs ranging from poor to moderate reliability (ICC = 0.17–0.71) (Supplementary Table S3, Figure S4-14).

### Associations between acid concentration changes and anxiety reductions

No statistically significant association between changes in SCFA concentrations and changes in LSAS-SR scores was found niether from the baseline to post-treatment, nor from the first to the 36-month follow-up (Supplementary Table S4, Figure S15-16).

### Exploratory analyses

Insomnia symptoms significantly decreased from pre- to (M ± SD = 9.33 ± 5.34) post-treatment (M ± SD = 6.67 ± 5.08; *P* < 0.001, Cohen’s d = 0.54), and the effect remained at the 36-month follow-up (M ± SD = 6.29 ± 5.18) (*P* = 0.82, Cohen’s *d* = 0.037; Supplementary Table S5, Figure S17). No statistically significant associations were found between changes in SCFA concentrations and changes in ISI scores, either from pre- to post-treatment (*r* = 0.005–0.318, *P*_FDR_ ≥ 0.34) or from post-treatment to the 36-month follow-up (*r* = 0.06–0.24, *P*_FDR_ ≥ 0.90). Further, no statistically significant associations were found between pre-treatment SCFA concentrations and pre-treatment LSAS-SR social anxiety symptoms, and all correlation coefficients were negligible to weak (*r* = 0.01–0.29, *P*_FDR_ ≥ 0.31). No statistically significant associations were found between pre-treatment SCFA concentrations and LSAS-SR treatment response, either at post-treatment (*r* = 0.002–0.14, *P*_FDR_ ≥ 0.88) or at the 36-month follow-up (*r* = 0.06–0.23, *P*_FDR_ ≥ 0.56).

## Discussion

This study examined longitudinal changes in plasma SCFA concentrations following CBT in individuals with SAD, as well as multiple baseline differences relative to healthy controls. Over the 36-month study period, we observed increases in butyric, isobutyric, propionic and valeric acid concentrations, all of which followed non-linear trajectories primarily explained by time rather than individual variability, indicating a delayed but potentially therapy-related shift in microbial metabolite profiles. At pre-treatment (i.e., at both baseline assessments), SAD patients had significantly lower isobutyric acid concentrations compared to healthy controls.

Butyric acid, in particular, is known for its beneficial effects on the intestinal barrier and its potent anti-inflammatory properties [43–46]. It may also influence brain function by reducing neuroinflammation, regulating neurotransmitter concentrations, and strengthening the integrity of the blood-brain barrier [47–50]. Supporting this, butyrate supplementation has been shown to improve behavioral and physiological abnormalities in individuals with autism spectrum disorder [51, 52]. Moreover, a therapy combining sodium butyrate and fluoxetine has demonstrated greater antidepressant-like efficacy compared to using fluoxetine alone [53]. Further, an increase in plasma butyric acid concentrations was observed in schizophrenia patients after initial treatment with risperidone [54]. Here, we found that SAD patients treated with a non-pharmacological psychosocial intervention also showed increases in butyric acid at long-term follow-up.

We also found that propionic acid increased after CBT for patients with SAD. Accumulating evidence suggests that propionic acid has significant anti-inflammatory properties [55–58]. Propionic acid also protects the blood-brain barrier by activating FFAR3 receptors on human brain endothelial cells, reducing inflammation and oxidative stress [11]. In a rodent model, oral administration of propionic acid alleviated depressive-like behaviors and ameliorated chronic stress-induced brain abnormalities by modulating intestinal flora composition and histone-3-related epigenetic mechanisms, suggesting its antidepressant potential [59].

Valeric acid has also been associated with a range of beneficial properties, including anticancer, antidiabetic, antihypertensive, anti-inflammatory, and immunomodulatory effects. It has been reported to influence molecular pathways involved in diseases such as Alzheimer’s disease, Parkinson’s disease, and epilepsy [60–62]. Here, we add that valeric acid may also be involved in patients’ response to a psychosocial intervention for anxiety disorders.

Changes in caproic, formic, succinic, and acetic acids followed more linear trajectories in our study. Formic acid increased greatly, while caproic and succinic acids showed moderate increases, and acetic acid remained unchanged. Isovaleric acid increased until post-treatment, but was the only acid to later decline below baseline values. Notably, SCFAs with more linear trajectories were generally those that changed less over time, and were more influenced by individual-level factors, with minimal contribution from the fixed-effect of time (and indirectly the intervention).

Our pre-treatment analyses revealed initial differences in gut microbiota-derived metabolites between SAD patients and healthy controls. Isobutyric acid concentrations were significantly lower in SAD patients compared to healthy controls (*P*_FDR_ = 0.006). Similar trends were observed for propionic and succinic acid (*P* ≤ 0.027), though these did not remain significant after multiple comparison corrections (*P*_FDR_ = 0.080). Notably, these three acids were among the SCFAs that increased after CBT. Lower concentrations of these acids in SAD patients would support existing hypotheses about gut microbiota dysbiosis in anxiety disorders [7, 63]. Jiang et al. [63] reported that patients with SAD exhibited reduced gut microbial diversity and a marked depletion of SCFA-producing genera such as *Faecalibacterium* and *Eubacterium rectale*. Consistent with our findings, reduced concentrations of propionic and succinic acids were also observed in patients with depression [16] and ADHD [14]. Notably, succinic acid is an intermediate metabolite in the fermentation towards propionic acid and derives also from the host’s mitochondrial activity [11, 55–59]. Plasma levels of isobutyric acid were reported to be lower in patients with anorexia nervosa than in healthy controls and exhibited a positive correlation with body mass index (BMI) [64]. In the present study, this group difference remained stable across two baseline assessments conducted 10 to 11 weeks apart, and isobutyric acid levels subsequently normalized and increased following CBT.

As previously reported from this cohort, CBT yielded a significant reduction in LSAS-SR scores over time, and the most pronounced improvement was observed immediately after treatment, followed by continued modest improvement during the long-term follow-up [28]. These findings are consistent with previous studies, confirming sustained and long-term effectiveness of internet-delivered CBT in the treatment of SAD [22–24, 26]. Although SCFA concentrations changed significantly over time, particularly in butyric, formic, isobutyric, propionic and valeric acids, these changes were not significantly associated with changes in social anxiety symptom severity. This may be because of the temporal discrepancy between psychological and metabolic changes. This lag effect could obscure potential associations in a cross-timepoint correlation analysis. It could also be due to the heterogeneity of SAD and measurement of noise. The clinical features vary between individuals, and SAD is also associated with considerable variability in personality traits [56]. Likewise, SCFAs are highly sensitive to dietary intake and individual metabolic differences. Fibre-rich diets, such as the mediterranean diet, are known to enhance SCFA production [65–67], which can, in turn, affect the gut microbiota composition and subsequently alter bacterial metabolite profiles. It is thus possible that symptom improvements in SAD patients may gradually lead to changes in both lifestyle and dietary habits.

While mixed models were applied to account for individual variability, the poor to moderate test-retest reliability observed for several SCFAs suggests measurement variability, which may have diluted real effects and reduced statistical power. Although the reliability of individual SCFA measures was modest, we observed a consistent and replicable difference in isobutyric acid concentration between patients and controls at two independent time points. This convergence of evidence supports the robustness of the group-level difference despite notable within-subject variability. Importantly, this pattern is consistent with a scenario in which within-individual measurements are noisy or state-dependent, yet systematic biological or pathological processes produce robust shifts at the group level.

To our knowledge, this is the first study to examine longitudinal changes in plasma SCFA concentrations before and after psychosocial treatment for any disorders. An important strength is that we investigated not only the immediate alterations in SCFA concentrations but also monitored these metabolite changes longitudinally for two years. We quantified SCFAs in plasma rather than faeces, providing a longitudinal readout of circulating, systemically available metabolites directly relevant to gut–brain signalling. This contributes to a better understanding of the microbiota-gut-brain axis in psychiatric conditions.

## Limitations

Long-term follow-ups with the healthy control group are lacking, as the study was statistically designed for within-patient group comparisons, but the study did utilize two baseline assessments to control the effects of time and regression to the mean in both patients and controls. Intrinsic to the study design, the samples from the last follow-up (4th follow-up) were stored at −80 °C for two years shorter than the other samples. However, we find this unlikely to explain the increased propionic, butyric, isobutyric and valeric acid concentrations in the fourth follow-up samples, relative to the first follow-up samples as (i) the other SCFAs did not increase from the first to the fourth follow-ups, (ii) SCFAs are reported stable for a few years at −80 °C [68], (iii) all samples had identical handling protocol when collected, and (iv) all samples were kept in sealed cryotubes and were freeze-thawed only once. The limited 10–11 week test–retest reliability of SCFA concentrations with ICCs ranging from poor to moderate (ICCs ranging from 0.17 to 0.71) suggests that SCFA concentrations may be influenced by transient or unmeasured factors such as recent dietary intake or metabolic fluctuations.

The absence of faecal samples precluded microbiome metagenomic profiling, and we were therefore unable to directly link plasma SCFAs alterations to specific microbial taxa or functional pathways. Prior studies have reported weak, non-significant, or even inverse correlations between faecal and circulating SCFAs levels [69, 70], highlighting the complex absorption and utilisation processes. Some of the gut-microbiome-derived SCFAs are consumed locally after absorption by the colonic epithelium, particularly butyrate. As the primary energy substrate for colonocytes, intracellular butyrate is rapidly oxidised via mitochondrial β-oxidation [71–73]. Following intestinal absorption, SCFAs are first transported via the portal vein to the liver, where a proportion undergoes hepatic metabolism before the remainder enters the systemic circulation. Consequently, faecal SCFAs primarily reflect microbial production within the gut lumen, whereas plasma SCFAs represent systemically available metabolites after epithelial utilisation and first-pass hepatic metabolism. Therefore, observed plasma SCFA changes are interpreted potentially as downstream metabolic readouts of host exposure and gut-brain signalling rather than direct proxies for microbial abundance or faecal production. This study did not include the assessments of dietary, physical activit, or other lifestyle-related factors, which may influence gut microbiota composition and SCFA production and may have changed gradually following anxiety symptom improvement. The observed SCFA alterations may reflect indirect effects of CBT mediated through lifestyle modifications rather than direct psychotherapeutic mechanisms. Participants fasted overnight before blood sampling, likely minimising the influence of dietary SCFAs absorbed in the small intestine. Future studies with dietary and lifestyle control and multi-omics integration are highly warranted to disentangle these interactions and assess the potential of microbiota-targeted interventions in SAD.

## Conclusions

This longitudinal study demonstrated that plasma concentrations increased in propionic, butyric, isobutyric and valeric acids over 2 years after SAD patients received effective CBT, and that plasma concentration of isobutyric acid was lower in SAD patients relative to matched healthy controls before treatment onset. Our findings suggest that the effects of a psychosocial intervention may involve a microbiota-associated biological pathway.

## Supplementary information

Supplemental Material

## Data Availability

The code used to perform the statistical analyses is publicly available at https://github.com/neuronssonlab/Cai_etal_2026_Transl_Psychiatry. Data supporting the findings of this study are available from the corresponding author upon reasonable request.
