# Supplementary material for: Plasma short-chain fatty acid concentrations in social anxiety disorder and changes after cognitive behavioral therapy
Source: Transl Psychiatry. 2026 Jun 3;16:295. doi: 10.1038/s41398-026-04134-y (PMC13233848; doi:10.1038/s41398-026-04134-y)
Supplement: Supplementary file 1 — Supplemental Material [file 41398_2026_4134_MOESM1_ESM.docx]

**Supplementary Materials**

**Plasma Short**-**Chain Fatty Acid Concentrations in Social Anxiety Disorder and Changes After Cognitive Behavioral Therapy**

Wenjie Cai,^1,2^ Miranda Stiernborg,^1,2^ Alexander Wolthon,^3,4^ Rikard Landberg,^5^ Amirhossein Manzouri,^3,6,7,8^ Tomas Furmark,^9^ Catharina Lavebratt,^1,2^ Kristoffer N. T. Månsson^6,7,8*^

**Affiliations**

^1^ Department of Molecular Medicine and Surgery, Karolinska Institutet, Stockholm, Sweden

^2^ Karolinska University Hospital Solna, Center for Molecular Medicine, Stockholm, Sweden

^3^ Department of Psychology, Stockholm University, Stockholm, Sweden

^4^ Department of Comparative Medicine, Karolinska Institutet, Stockholm, Sweden

^5^ Department of Life Sciences, Division of Food and Nutrition Science, Chalmers University of Technology, Gothenburg, Sweden

^6^ Department of Clinical Neuroscience, Karolinska Institutet, Stockholm, Sweden

^7^ Centre for Psychiatry Research, Department of Clinical Neuroscience, Karolinska Institutet, & Stockholm Health Care Services, Region Stockholm, Stockholm, Sweden

^8^ Department of Clinical Psychology and Psychotherapy, Babeș-Bolyai University, Cluj-Napoca, Romania

^9^ Department of Psychology, Uppsala University, Uppsala, Sweden

*Corresponding author: Kristoffer N. T. Månsson, kristoffer.mansson@ki.se, +46(0)70 5803267


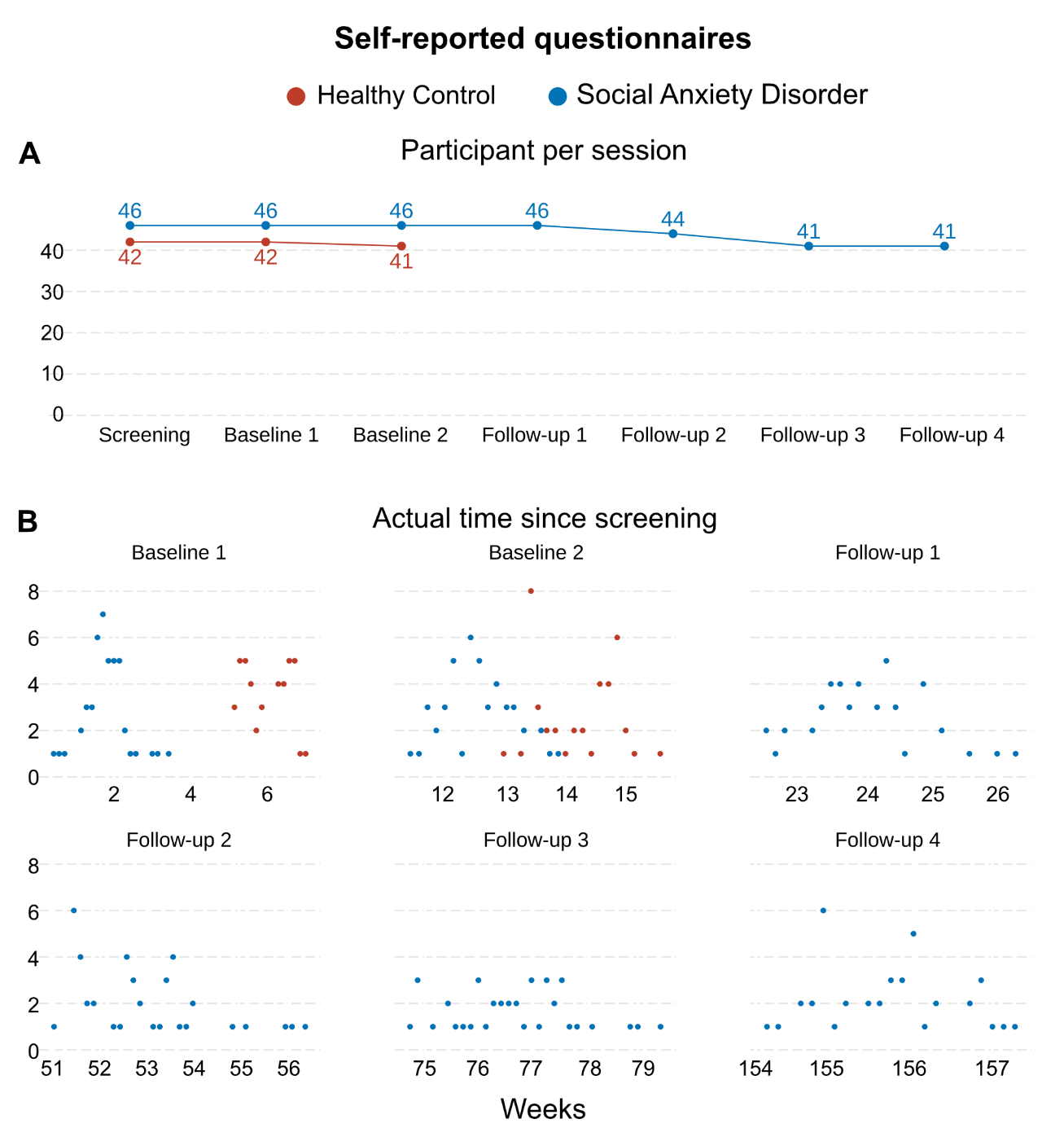


**Figure S1. Participation counts and dates of clinical assessment for social anxiety disorder patients and healthy controls. A** The number of participants at 6 time points. **B** Displays the time (in weeks) elapsed since initial screening for each participant. Y-axis represents the number of participants.


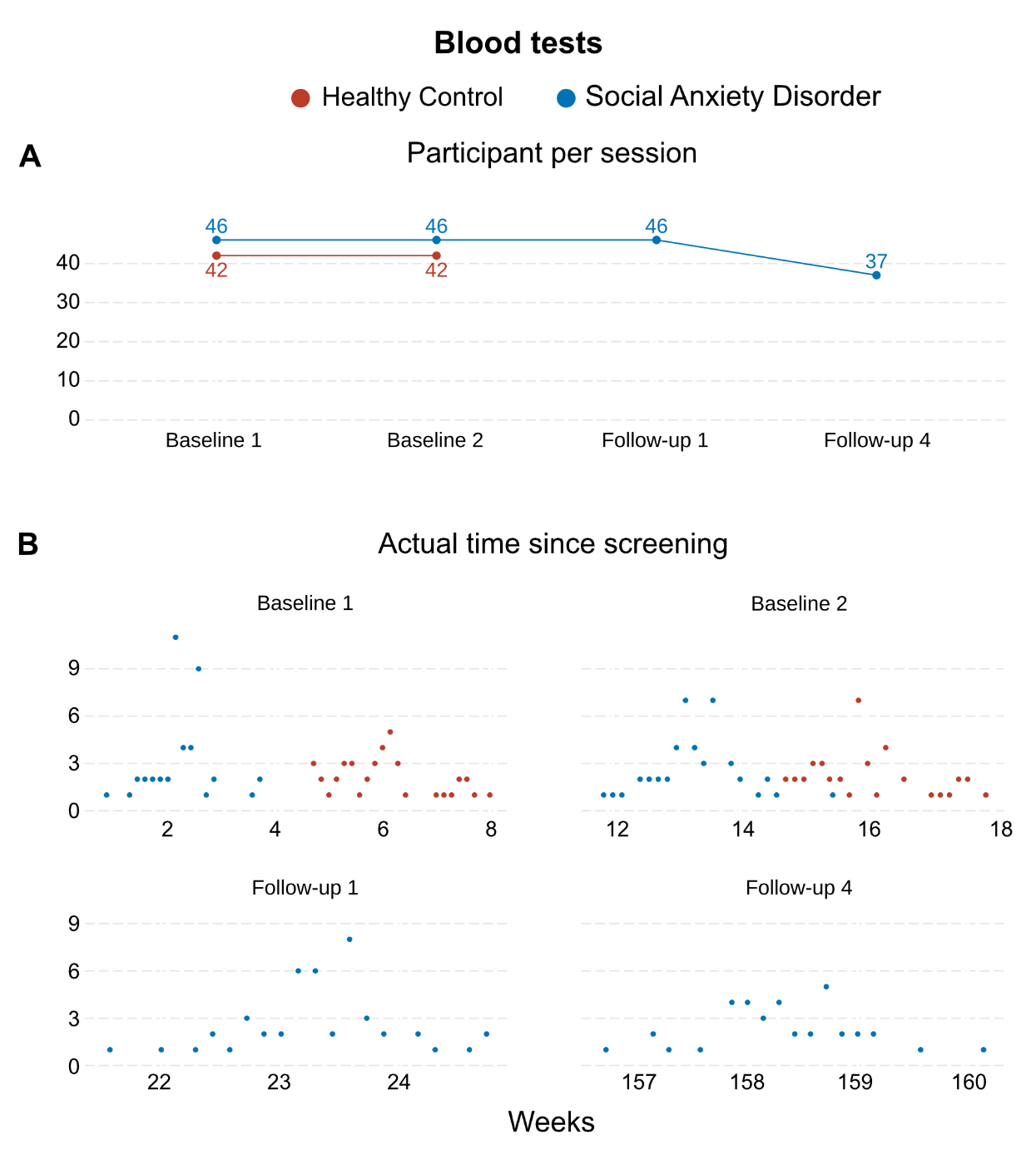


**Figure S2. Participation counts and dates of blood sampling for social anxiety disorder patients and healthy controls. A** The number of participants at 4 time points. **B** Displays the time (in weeks) elapsed since initial screening for each participant. Y-axis represents the number of participants.


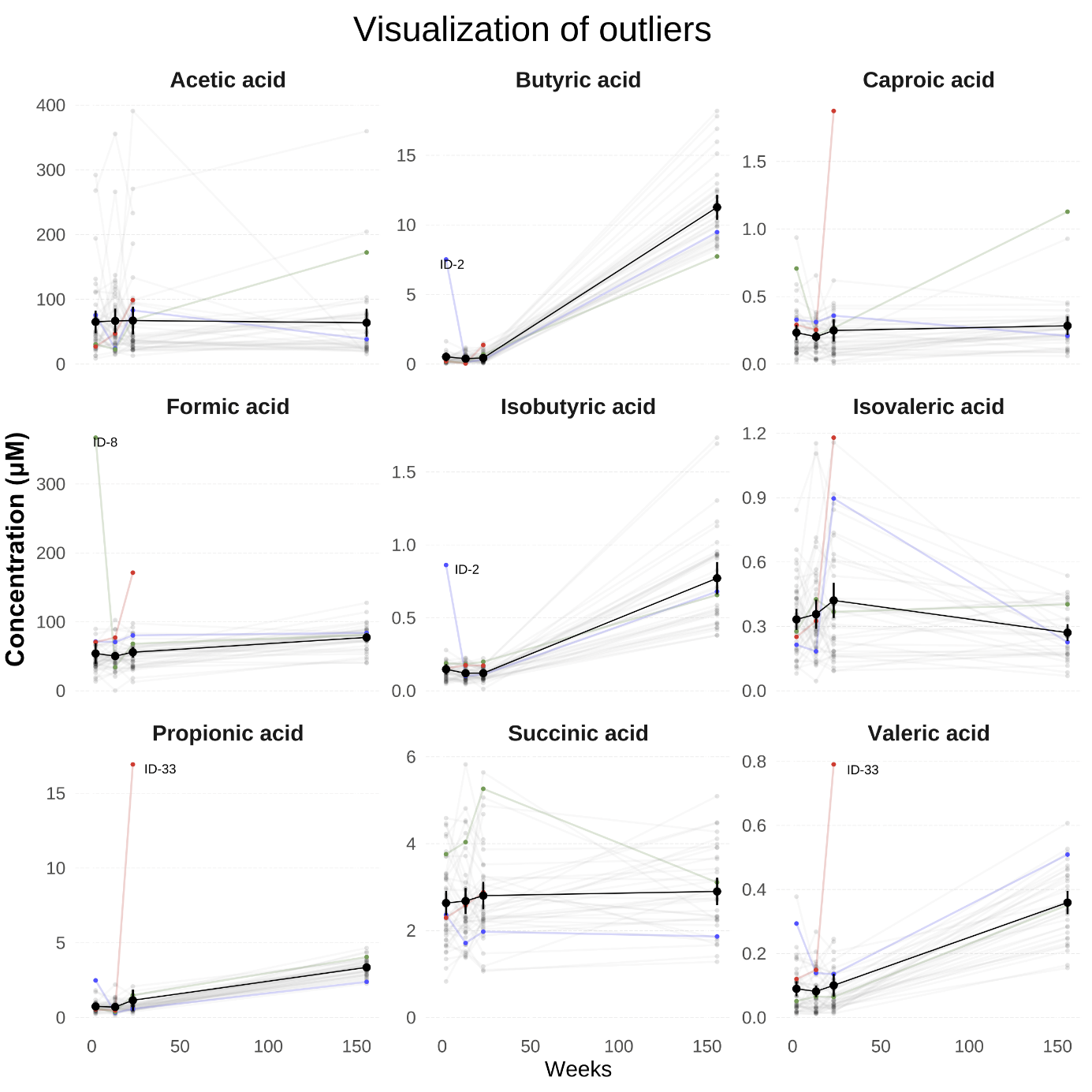


**Figure S3. Short-chain fatty acids change in patients with social anxiety disorder (SAD) across weeks including also outliers.** The concentration of each SCFA (μM) in SAD patients (outliers are included) over four time points: two baseline assessments (week 2 and week 13 after recruitment), first follow-up assessments at week 24, and the final assessment at week 156. Coloured lines (red, green and blue) represent three outliers. Error bars represent 95% CI.


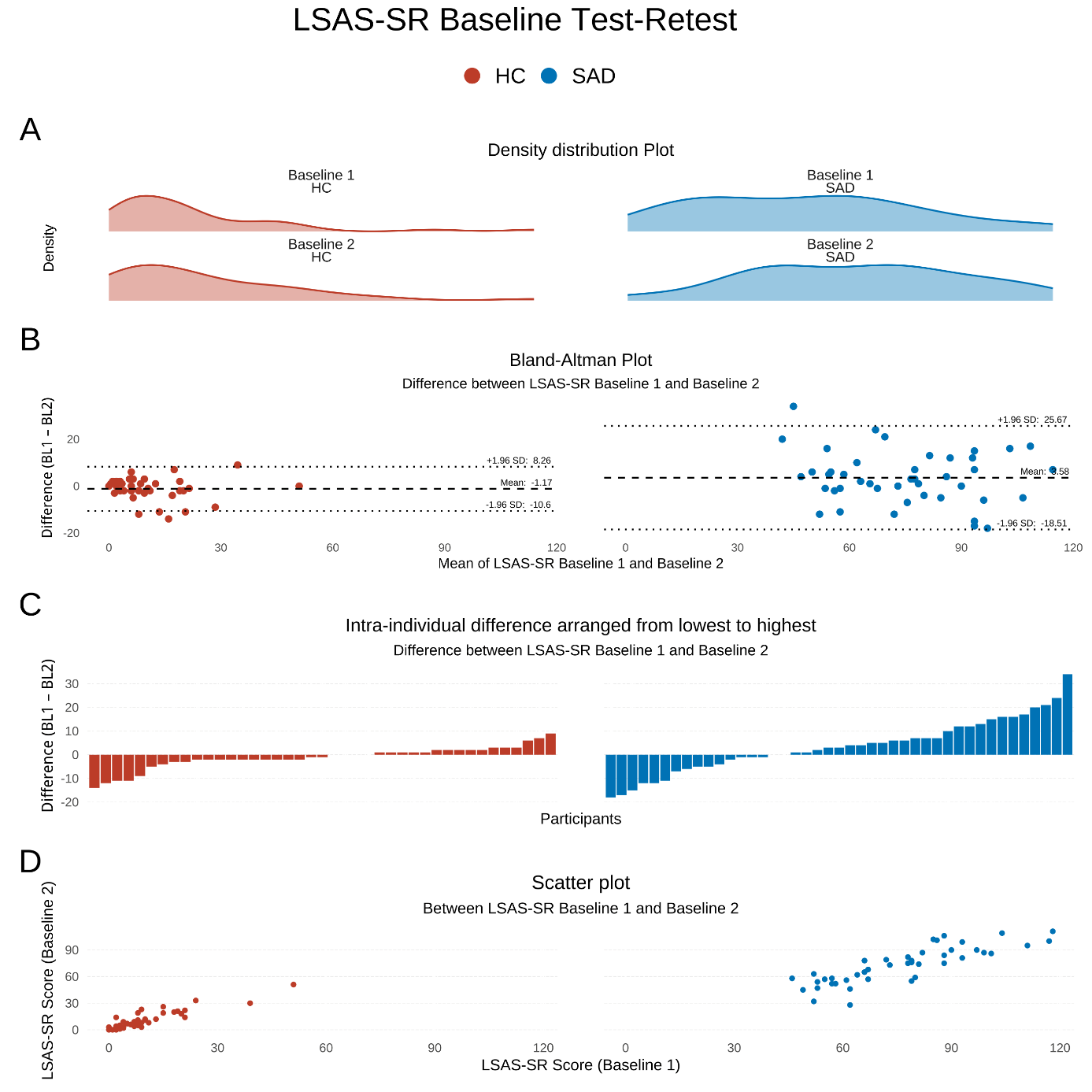
**Figure S4. Test-retest reliability of LSAS-SR score between two baseline assessments in social anxiety disorder (SAD) patients and healthy controls (HC). A)** Density distribution plots of LSAS-SR scores at Baseline 1 and Baseline 2 for each group. **B)** Bland-Altman plot visualises the agreement between the two baseline scores, with mean differences close to zero and 95% limits of agreement indicated by dashed lines. **C)** Intra-individual score differences (Baseline 1 – Baseline 2), ordered from lowest to highest, highlight minimal variation in HC and greater variability in the SAD group. **D)** Scatter plot of LSAS-SR scores at Baseline 1 vs. Baseline 2.


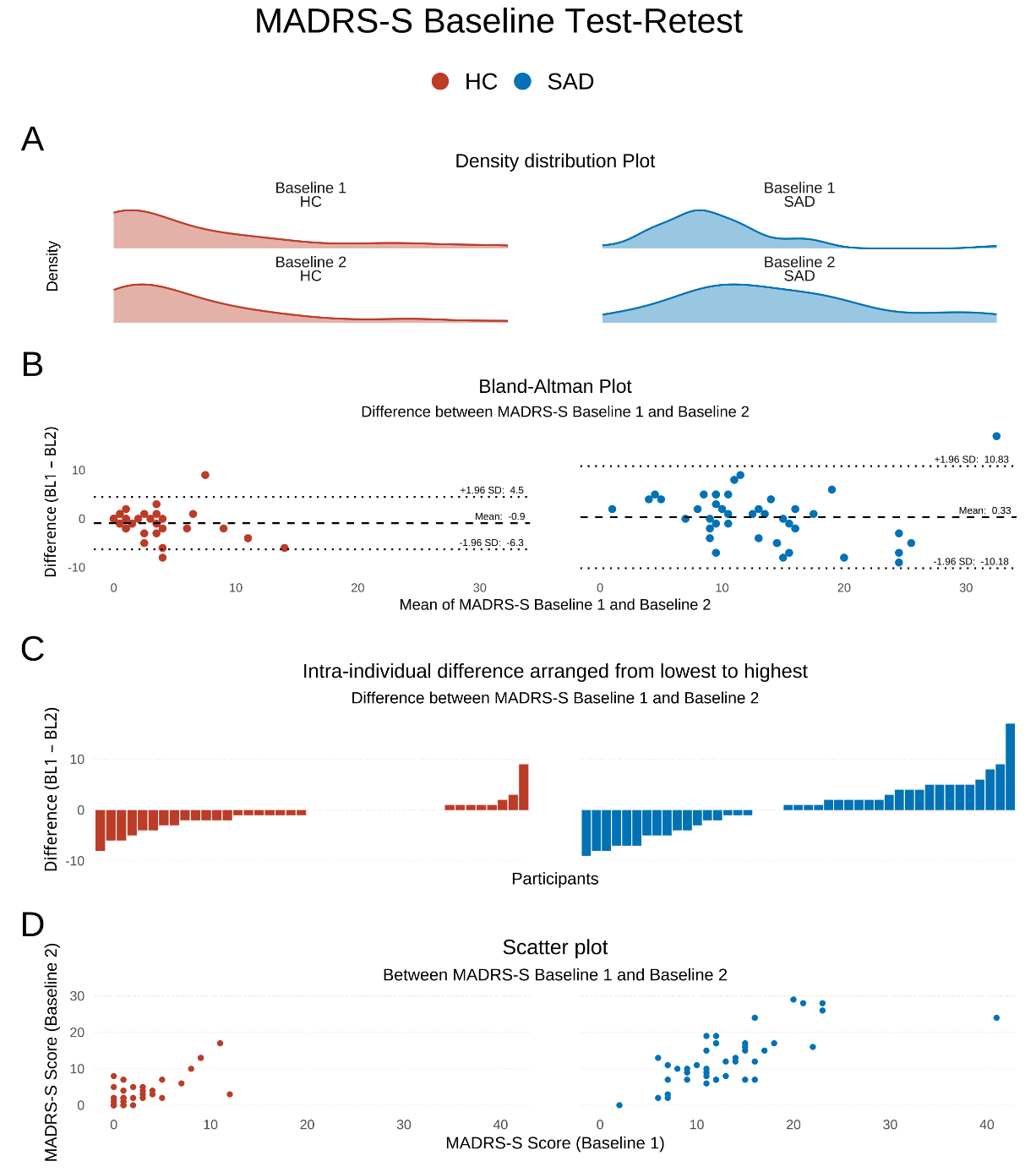


**Figure S5. Test-retest reliability of MADRS-S score between two baseline assessments in social anxiety disorder (SAD) patients and healthy controls (HC). A)** Density distribution plots of MADRS-S scores at Baseline 1 and Baseline 2 for each group. **B)** Bland-Altman plot visualises the agreement between the two baseline scores, with mean differences close to zero and 95% limits of agreement indicated by dashed lines. **C)** Intra-individual score differences (Baseline 1 – Baseline 2), ordered from lowest to highest, highlight minimal variation in HC and greater variability in the SAD group. **D)** Scatter plot of MADRS-S scores at Baseline 1 vs. Baseline 2.


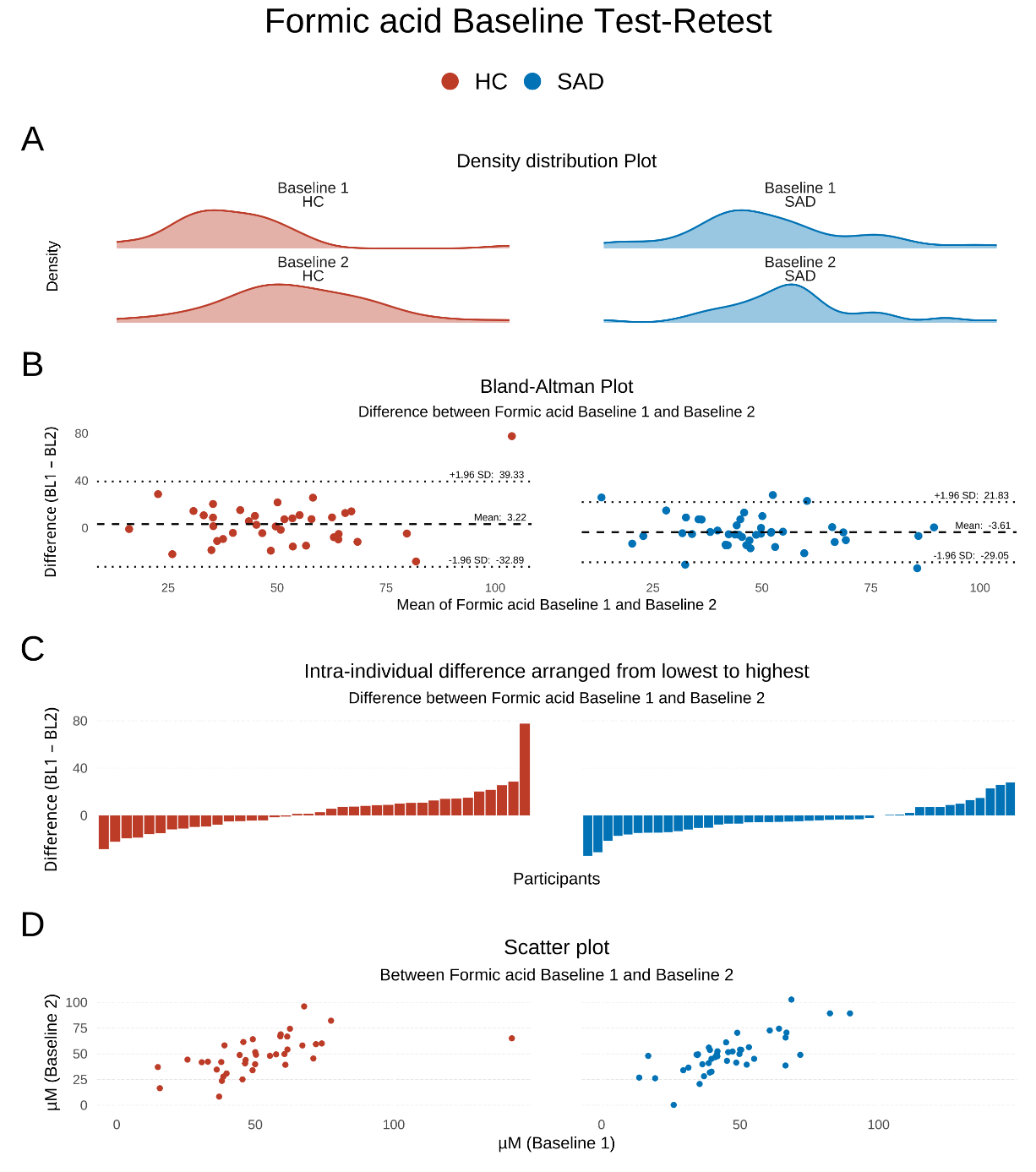


**Figure S6. Test-retest reliability of plasma formic acid levels between two baseline assessments in social anxiety disorder (SAD) patients and healthy controls (HC). A** Density distribution plot showing the formic acid concentration at Baseline 1 and Baseline 2 for each group. **B** Bland-Altman plot visualises the agreement between the two baseline levels, with mean differences close to zero and 95% limits of agreement indicated by dashed lines. **C** Intra-individual level differences (Baseline 1 – Baseline 2), ordered from lowest to highest. **D** Scatter plot of formic acid at Baseline 1 vs. Baseline 2.


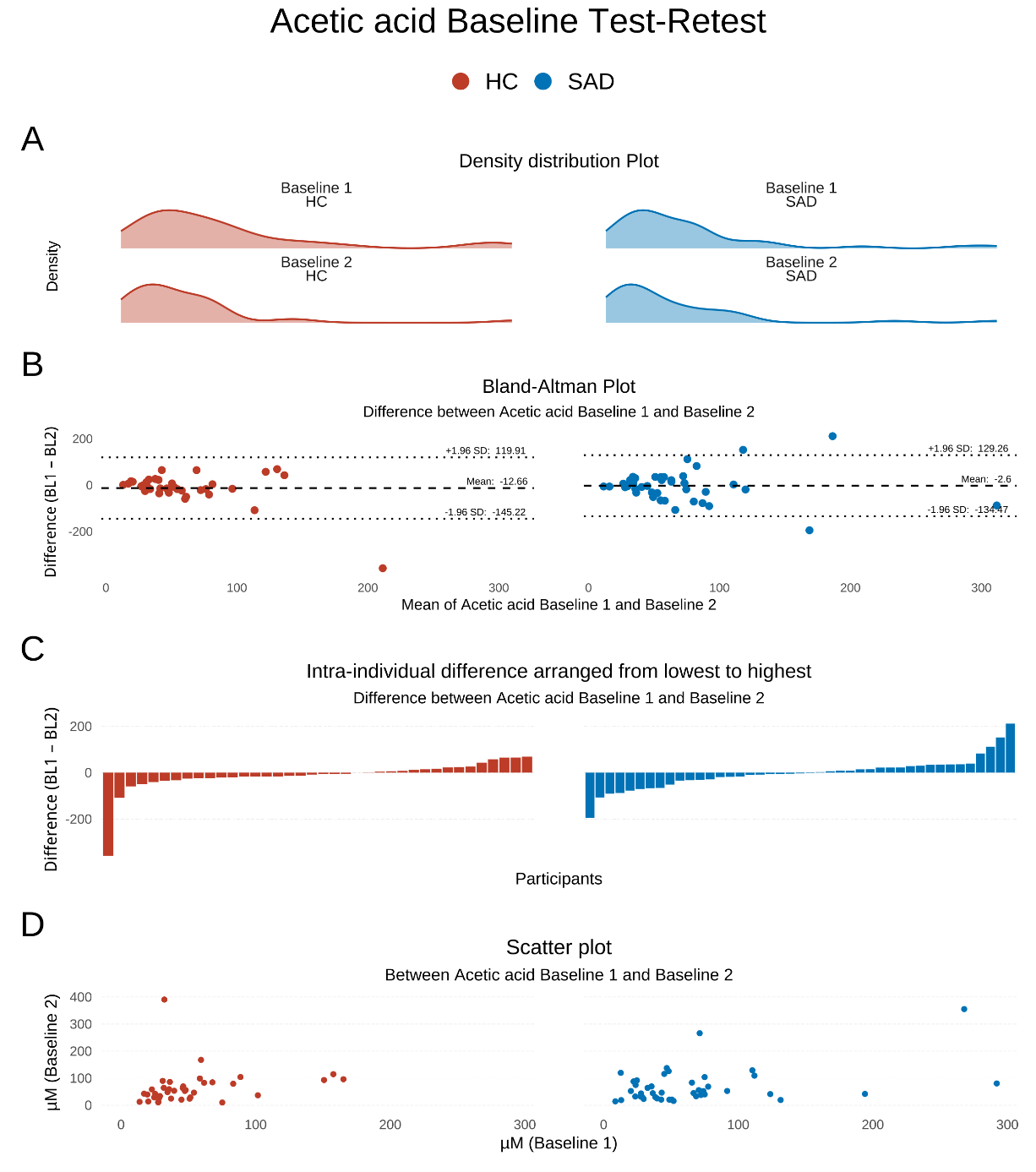


**Figure S7. Test-retest reliability of plasma acetic acid levels between two baseline assessments in social anxiety disorder (SAD) patients and healthy controls (HC). A** Density distribution plot showing the acetic acid concentration at Baseline 1 and Baseline 2 for each group. **B** Bland-Altman plot visualises the agreement between the two baseline levels, with mean differences close to zero and 95% limits of agreement indicated by dashed lines. **C** Intra-individual level differences (Baseline 1 – Baseline 2), ordered from lowest to highest. **D** Scatter plot of acetic acid at Baseline 1 vs. Baseline 2.


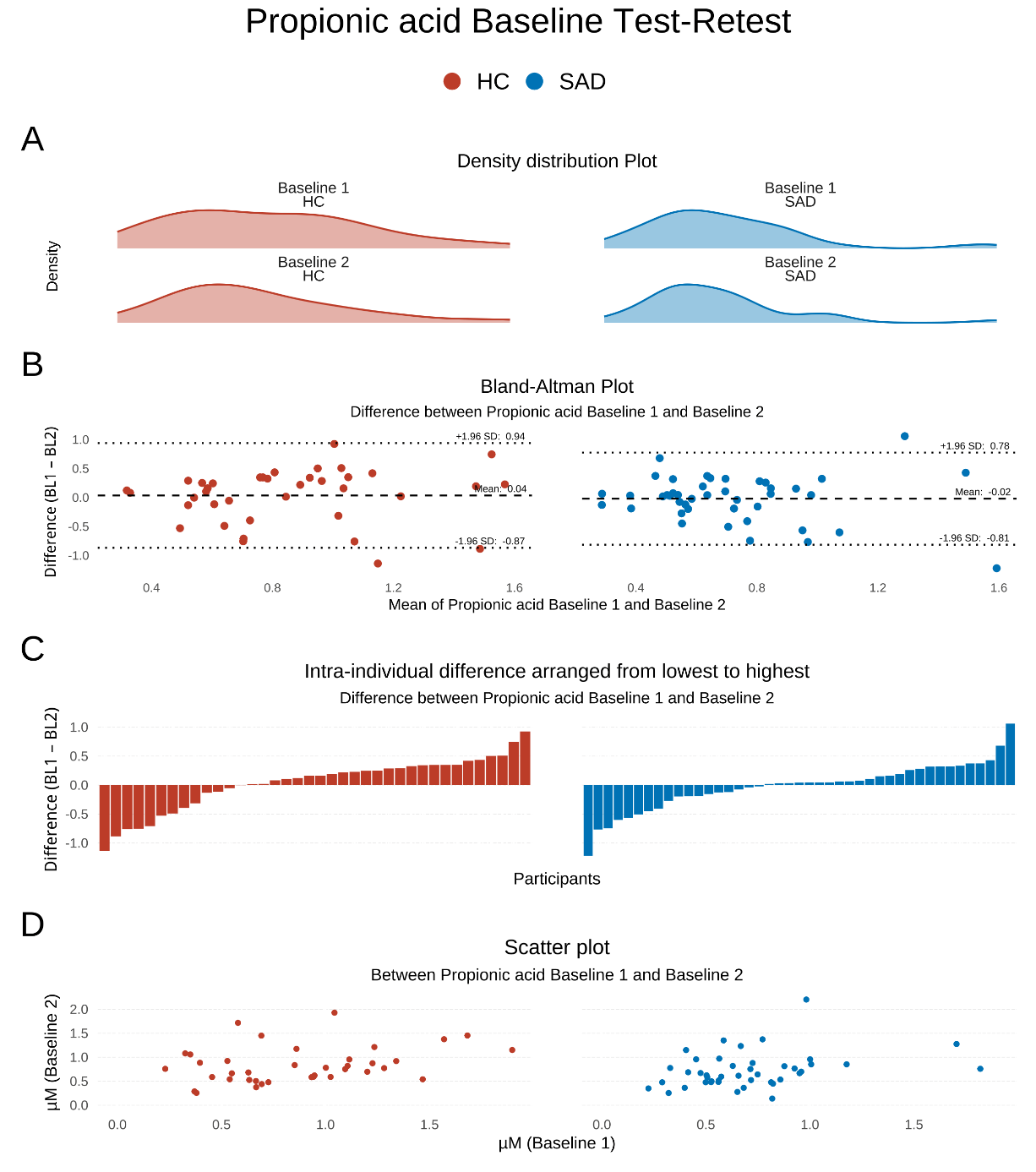


**Figure S8. Test-retest reliability of plasma propionic acid levels between two baseline assessments in social anxiety disorder (SAD) patients and healthy controls (HC). A** Density distribution plot showing the propionic acid concentration at Baseline 1 and Baseline 2 for each group. **B** Bland-Altman plot visualises the agreement between the two baseline levels, with mean differences close to zero and 95% limits of agreement indicated by dashed lines. **C** Intra-individual level differences (Baseline 1 – Baseline 2), ordered from lowest to highest. **D** Scatter plot of propionic acid at Baseline 1 vs. Baseline 2.


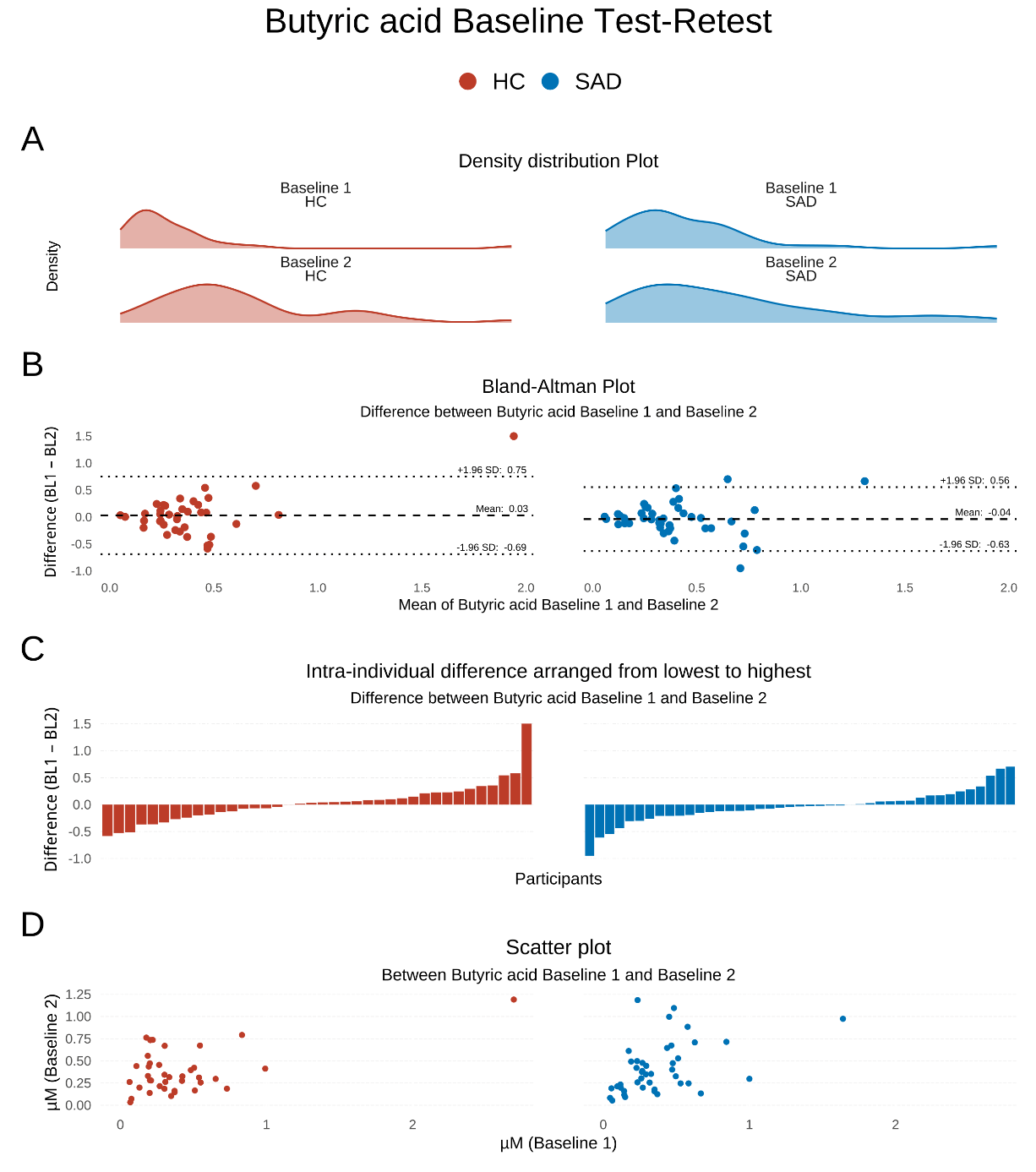


**Figure S9. Test-retest reliability of plasma** **butyric acid levels between two baseline assessments in social anxiety disorder (SAD) patients and healthy controls (HC). A** Density distribution plot showing the butyric acid concentration at Baseline 1 and Baseline 2 for each group. **B** Bland-Altman plot visualises the agreement between the two baseline levels, with mean differences close to zero and 95% limits of agreement indicated by dashed lines. **C** Intra-individual level differences (Baseline 1 – Baseline 2), ordered from lowest to highest. **D** Scatter plot of butyric acid at Baseline 1 vs. Baseline 2.


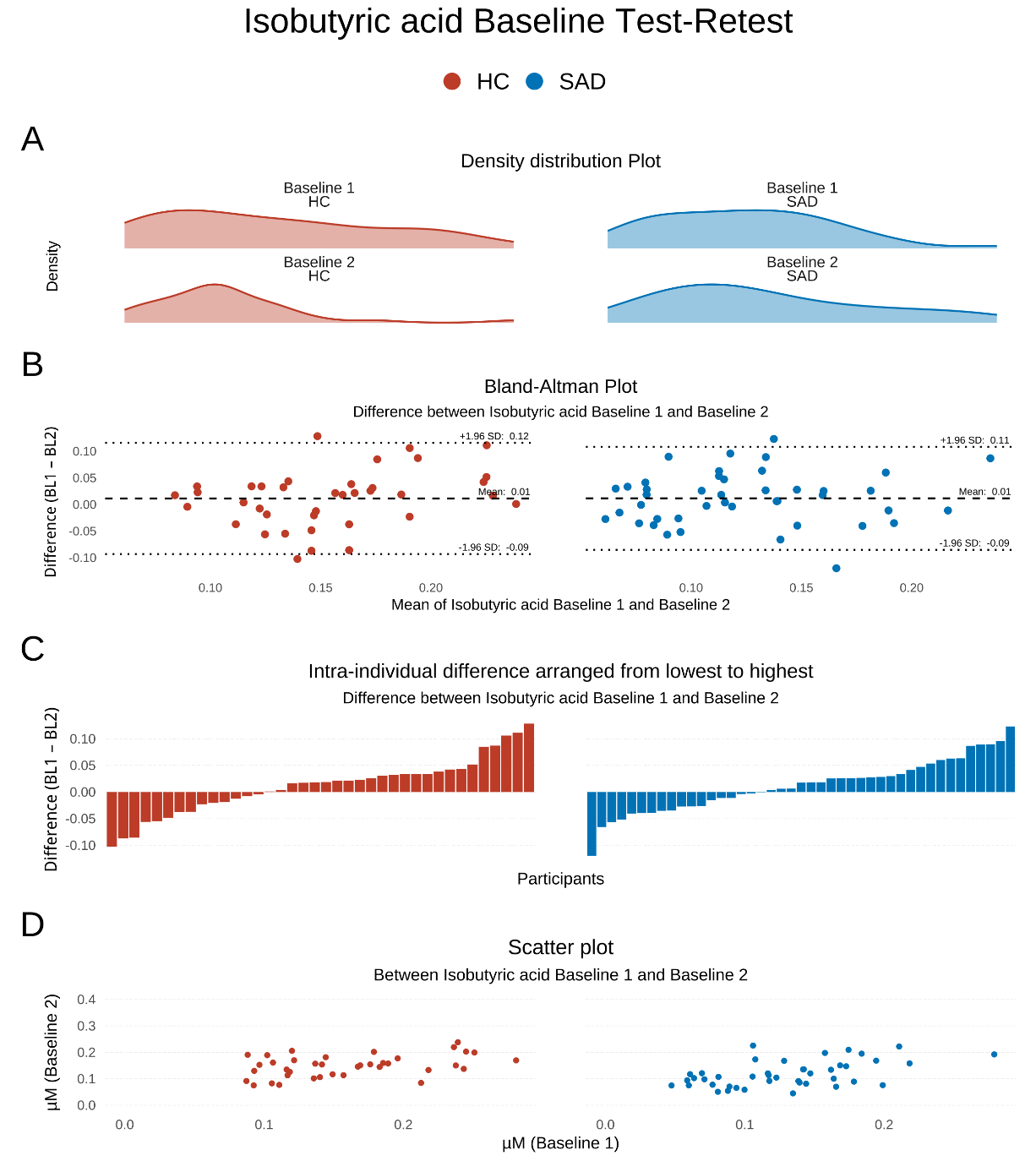


**Figure S10. Test-retest reliability of plasma isobutyric acid levels between two baseline assessments in social anxiety disorder (SAD) patients and healthy controls (HC). A** Density distribution plot showing the isobutyric acid concentration at Baseline 1 and Baseline 2 for each group. **B** Bland-Altman plot visualises the agreement between the two baseline levels, with mean differences close to zero and 95% limits of agreement indicated by dashed lines. **C** Intra-individual level differences (Baseline 1 – Baseline 2), ordered from lowest to highest. **D** Scatter plot of isobutyric acid at Baseline 1 vs. Baseline 2.


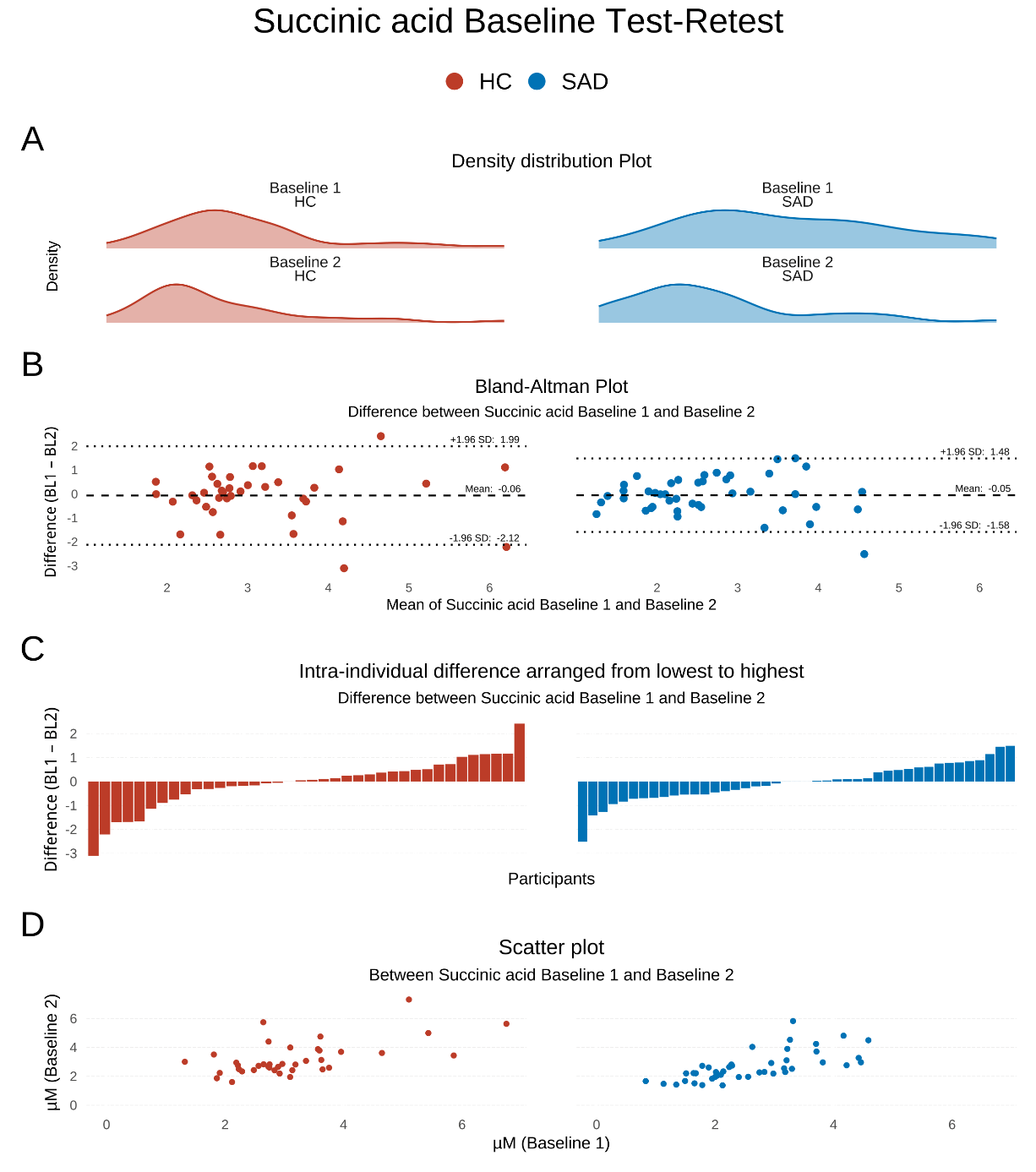


**Figure S11. Test-retest reliability of plasma succinic acid levels between two baseline assessments in social anxiety disorder (SAD) patients and healthy controls (HC). A** Density distribution plot showing the succinic acid concentration at Baseline 1 and Baseline 2 for each group. **B** Bland-Altman plot visualises the agreement between the two baseline levels, with mean differences close to zero and 95% limits of agreement indicated by dashed lines. **C** Intra-individual level differences (Baseline 1 – Baseline 2), ordered from lowest to highest. **D** Scatter plot of succinic acid at Baseline 1 vs. Baseline 2.


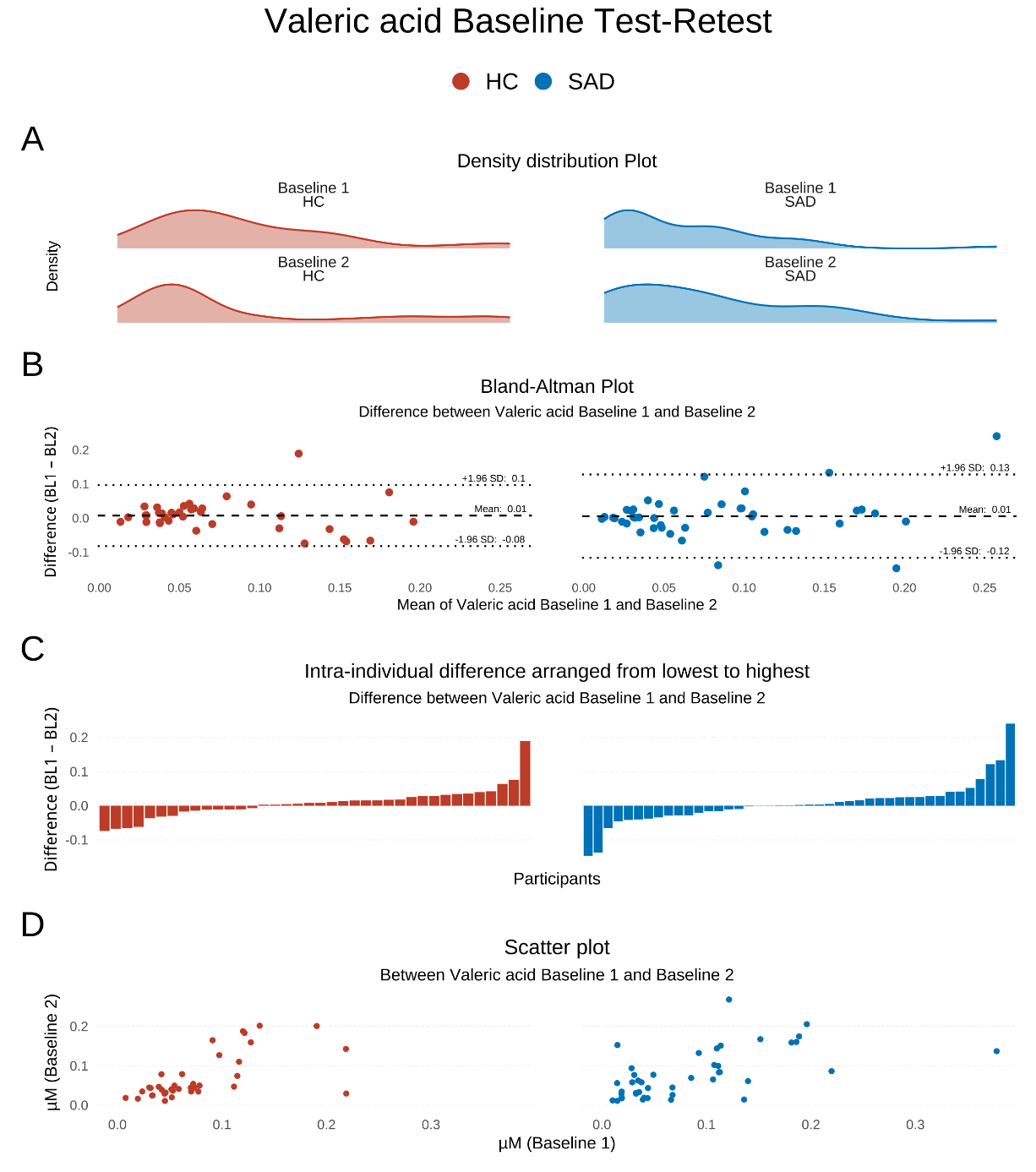


**Figure S12. Test-retest reliability of plasma valeric acid levels between two baseline assessments in social anxiety disorder (SAD) patients and healthy controls (HC). A** Density distribution plot showing the valeric acid concentration at Baseline 1 and Baseline 2 for each group. **B** Bland-Altman plot visualises the agreement between the two baseline levels, with mean differences close to zero and 95% limits of agreement indicated by dashed lines. **C** Intra-individual level differences (Baseline 1 – Baseline 2), ordered from lowest to highest. **D** Scatter plot of valeric acid at Baseline 1 vs. Baseline 2.


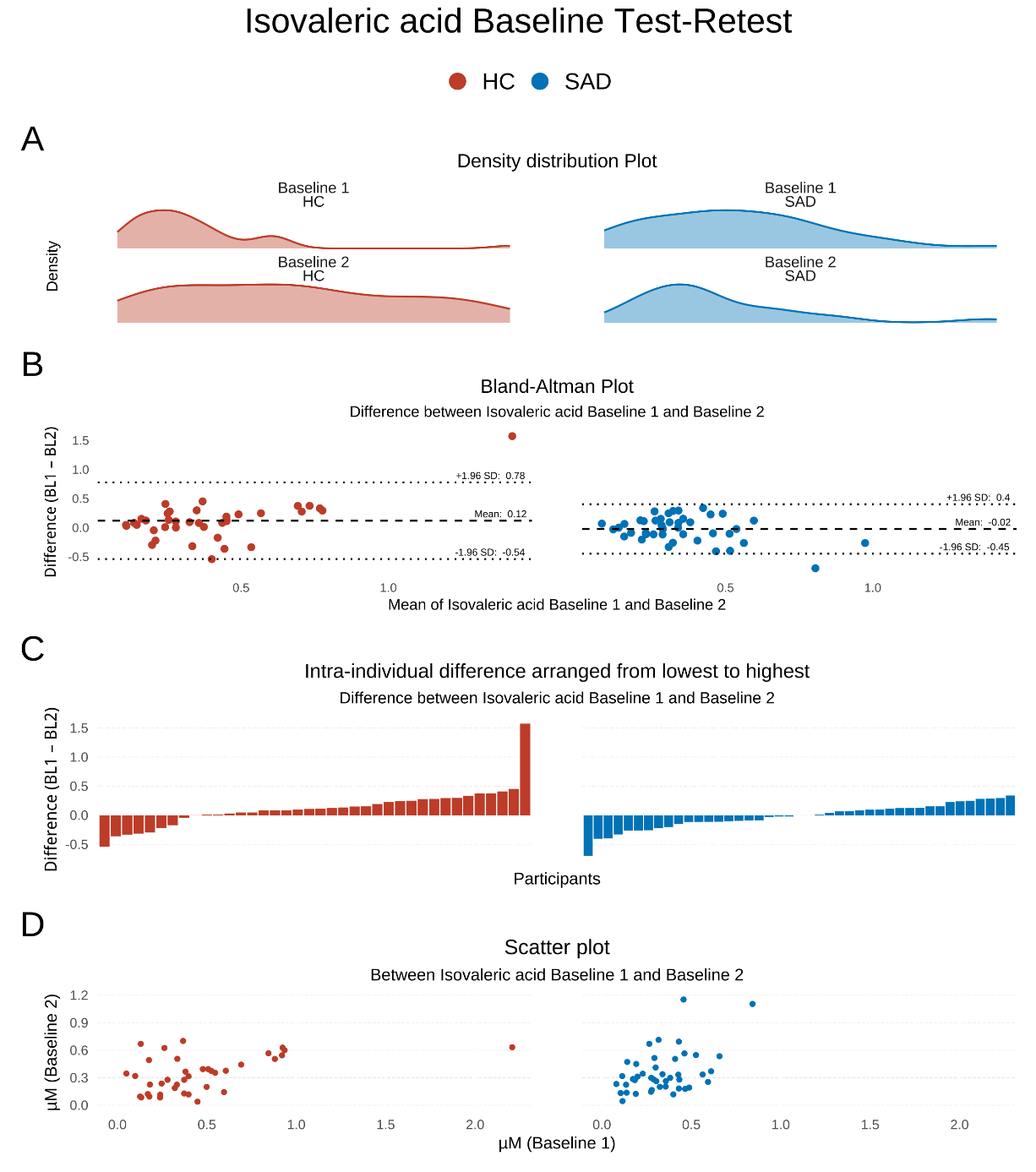


**Figure S13. Test-retest reliability of plasma** **isovaleric acid levels between two baseline assessments in social anxiety disorder (SAD) patients and healthy controls (HC). A** Density distribution plot showing the isovaleric acid concentration at Baseline 1 and Baseline 2 for each group. **B** Bland-Altman plot visualises the agreement between the two baseline levels, with mean differences close to zero and 95% limits of agreement indicated by dashed lines. **C** Intra-individual level differences (Baseline 1 – Baseline 2), ordered from lowest to highest. **D** Scatter plot of isovaleric acid at Baseline 1 vs. Baseline 2.


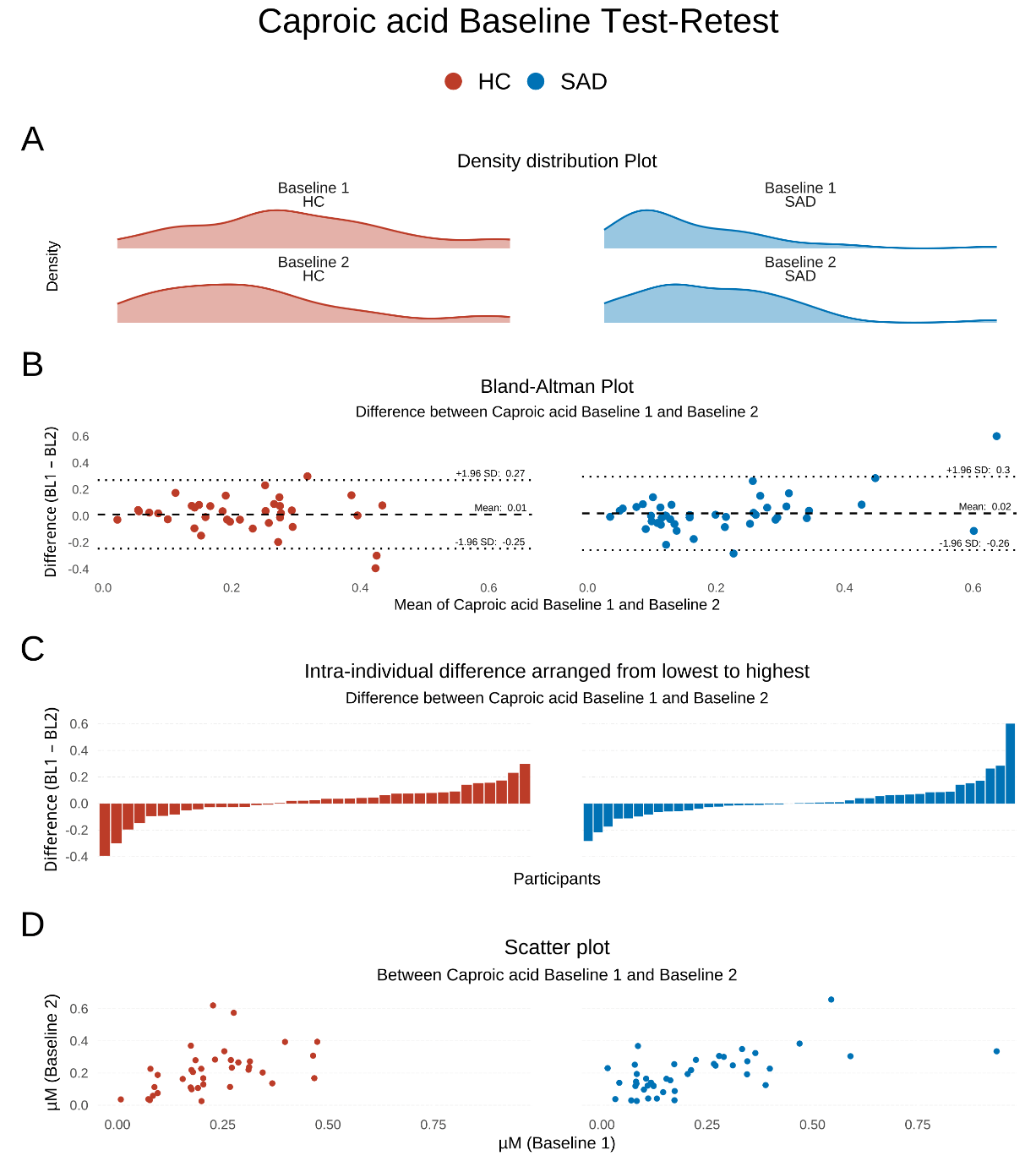


**Figure S14. Test-retest reliability of plasma caproic acid levels between two baseline assessments in social anxiety disorder (SAD) patients and healthy controls (HC). A** Density distribution plot showing the caproic acid concentration at Baseline 1 and Baseline 2 for each group. **B** Bland-Altman plot visualises the agreement between the two baseline levels, with mean differences close to zero and 95% limits of agreement indicated by dashed lines. **C** Intra-individual level differences (Baseline 1 – Baseline 2), ordered from lowest to highest. **D** Scatter plot of caproic acid at Baseline 1 vs. Baseline 2.


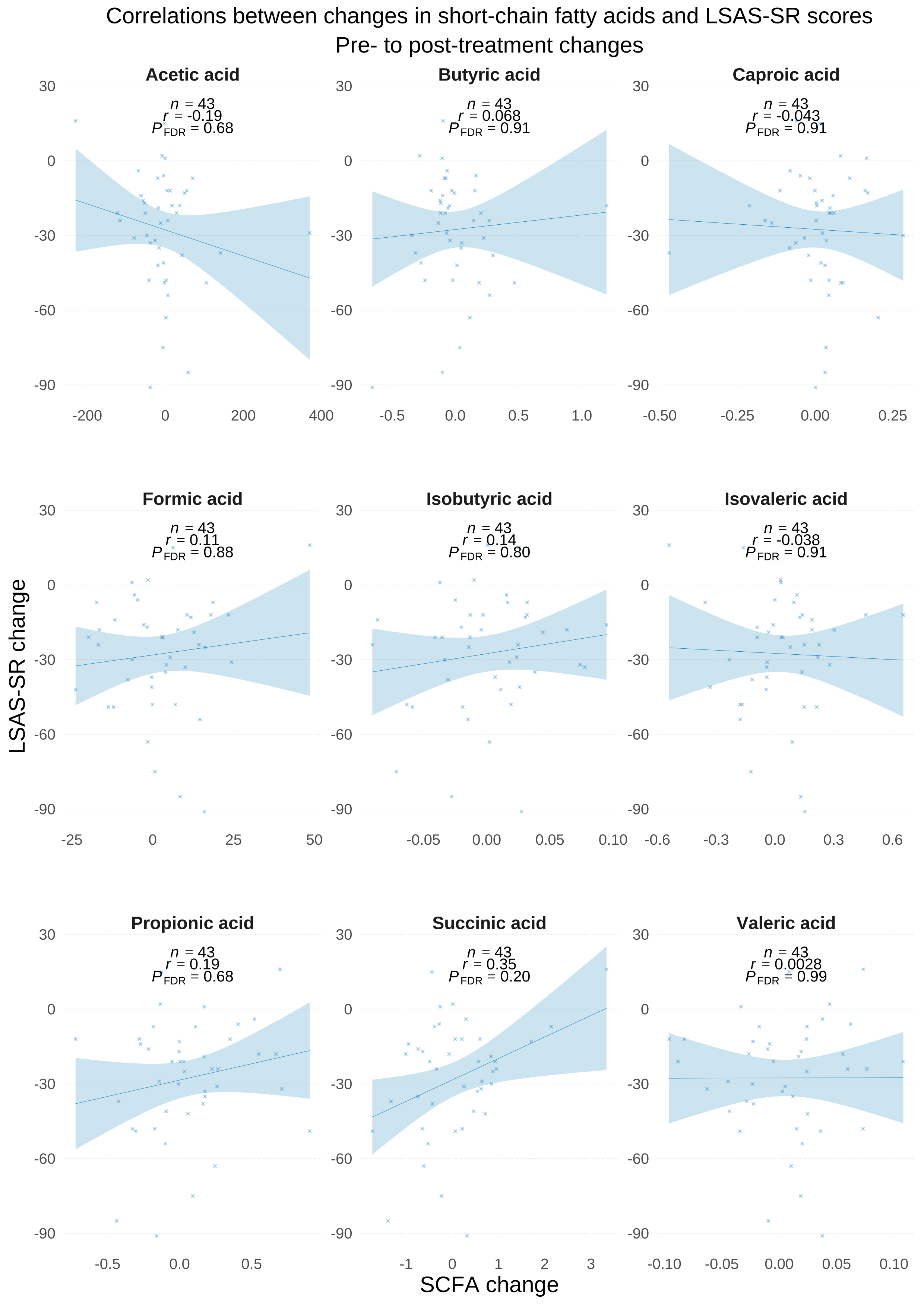


**Figure S15. Correlations between changes in short-chain fatty acids (SCFAs) and social anxiety symptoms (i.e., LSAS-SR scores).** The correlation between changes in plasma levels of individual SCFAs and changes in LSAS-SR scores (the second baseline assessment to the first follow-up assessment) in patients with social anxiety disorder (n = 43). Each figure shows a scatter plot with a linear regression line and 95% confidence interval (shaded area).

**Abbreviations:** LSAS-SR, Liebowitz Social Anxiety Scale–Self report version


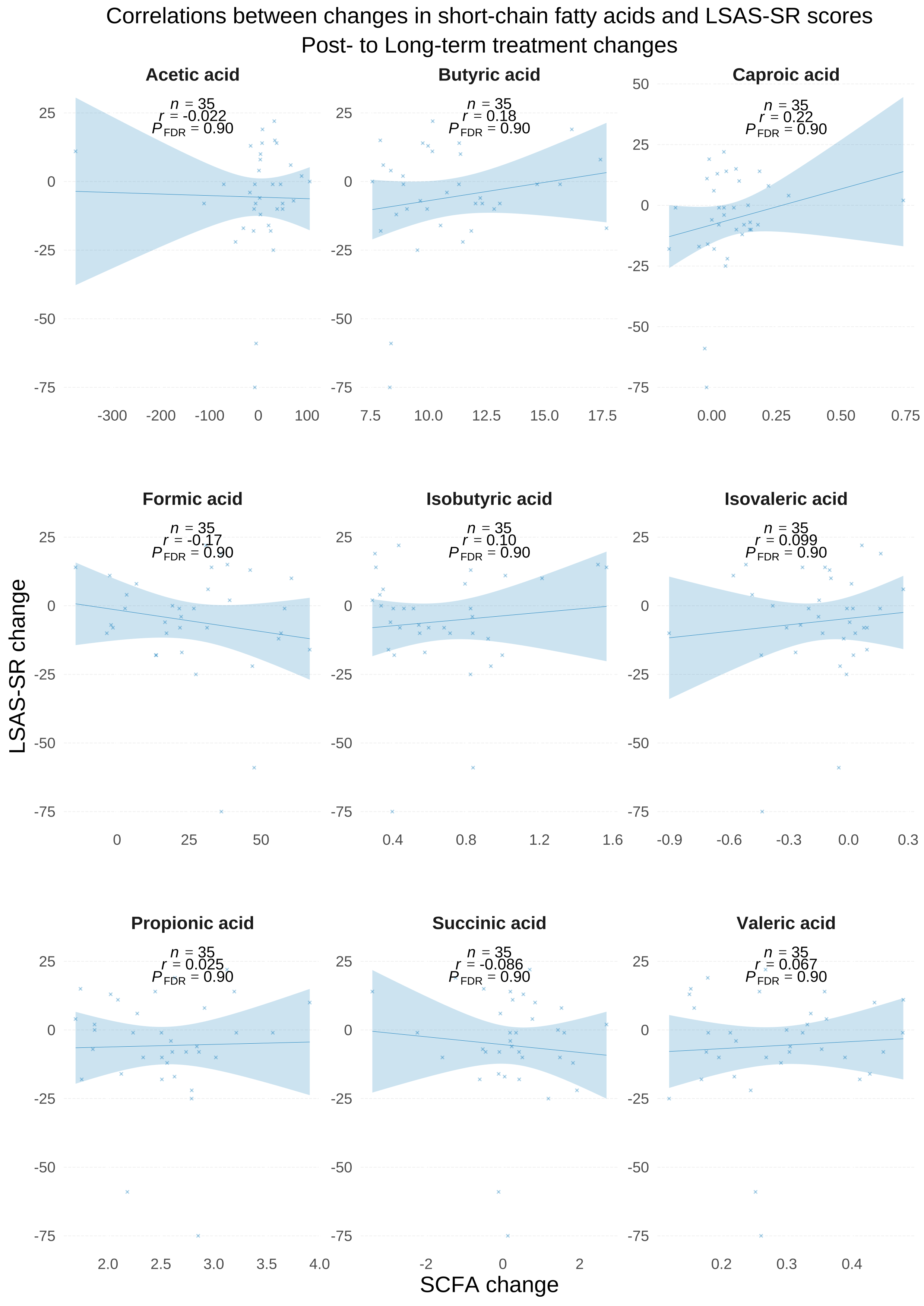


**Figure S16. Correlations between changes in short-chain fatty acids (SCFAs) and social anxiety symptoms (i.e., LSAS-SR scores).** The correlation between changes in plasma levels of individual SCFAs and changes in LSAS-SR scores (the first to the last follow-up assessment) in patients with social anxiety disorder (n = 35). Each figure shows a scatter plot with a linear regression line and 95% confidence interval (shaded area).

**Abbreviations:** LSAS-SR, Liebowitz Social Anxiety Scale–Self report version.


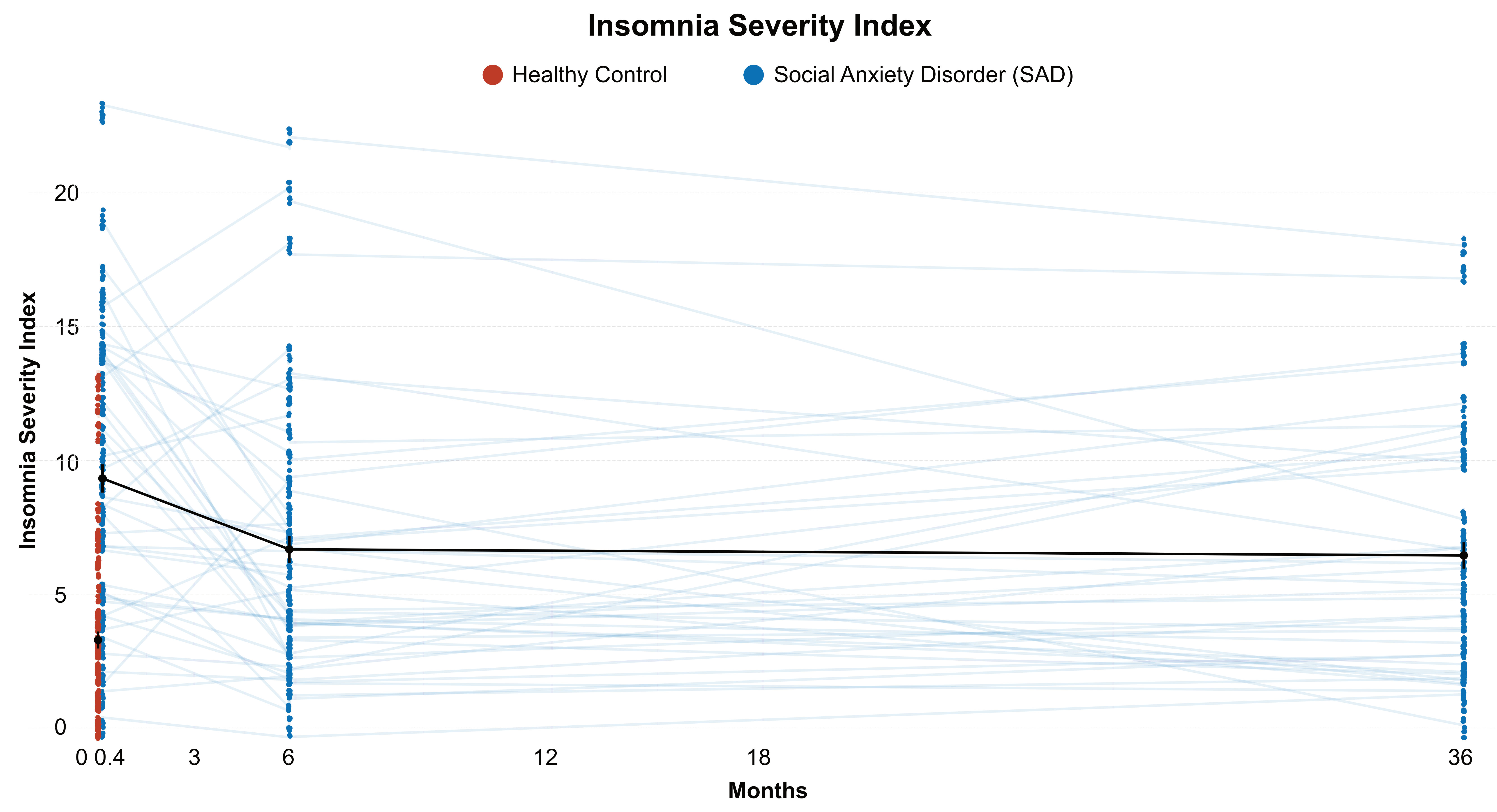


**Figure S17. Longitudinal changes in insomnia severity in patients with social anxiety disorder and healthy controls.**
Insomnia Severity Index (ISI) scores at baseline, 6 months, and 36 months in patients with social anxiety disorder (SAD; blue) and healthy controls (red). Individual trajectories are shown as faint lines connecting repeated measures within participants. Points represent observed values at each timepoint. The solid black line represents the group-level mean trajectory for SAD patients across time. Error bars represent 95% CI.

**Table S1.** Generalized additive mixed models examining longitudinal changes in short-chain fatty acids and clinical scores in social anxiety disorder patients.

|  | **Fixed effect (time)** | | | | | **Random effect (participant)** | | | | **Other model statistics** | | | | | | |
| --- | --- | --- | --- | --- | --- | --- | --- | --- | --- | --- | --- | --- | --- | --- | --- | --- |
|  | **Edf** | **Ref. df** | **F** | ***P*** | ***P_FDR_*** | **Edf** | **F** | ***P*** | ***P_FDR_*** | **Deviance explained** | **Scale est** | **n** | **Nakagawa Marginal R2 (fixed effect)** | **Nakawaga 'random effect'** | **Nakagawa Conditional R2 (fixed & random effect)** | **Singular** |
| **Short-chain fatty acids** | | | | | | | | | | | | | | | |  |
| Acetic acid | 1.00 | 1.00 | 0.002 | 0.96 | 0.96 | 30.78 | 2.37 | <1e-16 | <1e-16 | 49.4 | 2709.06 | 164 | 2.695e-06 |  |  | True |
| Butyric acid | 2.07 | 2.26 | 936.38 | <1e-16 | <1e-16 | 0.28 | 0.007 | 0.47 | 0.47 | 93.0 | 1.58 | 164 | 0.888 | <1e-16 | 0.888 | False |
| Caproic acid | 1.20 | 1.36 | 10.53 | 4.29e-04 | 6.44e-04 | 36.30 | 6.53 | <1e-16 | <1e-16 | 71.8 | 0.008 | 164 | 0.029 | 0.612 | 0.641 | False |
| Formic acid | 1.00 | 1.00 | 186.16 | <1e-16 | <1e-16 | 36.30 | 5.69 | <1e-16 | <1e-16 | 77.8 | 132.73 | 164 | 0.542 |  |  | True |
| Isobutyric acid | 1.93 | 2.13 | 229.52 | <1e-16 | <1e-16 | 10.47 | 0.34 | 0.097 | 0.11 | 77.4 | 0.02 | 164 | 0.598 | 0.033 | 0.631 | False |
| Isovaleric acid | 1.78 | 2.01 | 6.54 | 0.002 | 0.003 | 30.86 | 2.92 | <1e-16 | <1e-16 | 56.3 | 0.02 | 164 | 3.573e-04 | 0.432 | 0.432 | False |
| Propionic acid | 2.00 | 2.19 | 770.77 | <1e-16 | <1e-16 | 26.43 | 1.80 | 2.90e-06 | 3.71e-06 | 93.2 | 0.11 | 164 | 0.821 | 0.057 | 0.878 | False |
| Succinic acid | 1.00 | 1.00 | 6.22 | 0.014 | 0.016 | 34.5 | 4.28 | <1e-16 | <1e-16 | 63.0 | 0.47 | 164 | 0.038 |  |  | True |
| Valeric acid | 1.98 | 2.17 | 320.71 | <1e-16 | <1e-16 | 33.26 | 3.4 | <1e-16 | <1e-16 | 86.7 | 0.003 | 164 | 0.547 | 0.218 | 0.765 | False |
|  | | | | | | | | | | | | | | | |  |
| **Clinical measures** | | | | | | | | | | | | | | | |  |
| LSAS-SR | 2.94 | 3.0 | 128.03 | <1e-16 |  | 37.15 | 7.52 | <1e-16 |  | 75.20% | 226.51 | 293 | 0.612 | 0.230 | 0.842 | False |
| MADRS-S | 2.89 | 2.99 | 29.06 | <1e-16 |  | 37.72 | 7.85 | <1e-16 |  | 64.90% | 21.99 | 291 | 0.277 | 0.439 | 0.716 | False |

**Table S2.** Differences between Social Anxiety Disorder (SAD) and healthy controls at baseline

|  | **Healthy controls**  **(Mean ± SD)** | **SAD patients**  **(Mean ± SD)** | **t** | **DoF** | ***P*** | ***P_FDR_*** | **Cohen's *d*** |
| --- | --- | --- | --- | --- | --- | --- | --- |
| **Short-chain fatty acids** | | | | | | | |
| Acetic acid | 61.20 ± 53.64 | 67.92 ± 62.23 | -0.62 | 82.20 | 0.535 | 0.671 | 0.06 |
| Butyric acid | 0.40 ± 0.35 | 0.39 ± 0.29 | 0.09 | 83.33 | 0.924 | 0.927 | 0.01 |
| Caproic acid | 0.22 ± 0.13 | 0.21 ± 0.15 | 0.53 | 80.53 | 0.597 | 0.671 | 0.05 |
| Formic acid | 50.07 ± 19.26 | 47.85 ± 17.55 | 0.61 | 83.92 | 0.540 | 0.671 | 0.06 |
| Isobutyric acid | 0.16 ± 0.06 | 0.12 ± 0.05 | 3.54 | 73.48 | < 0.001 | 0.006 | 0.34 |
| Isovaleric acid | 0.39 ± 0.30 | 0.35 ± 0.20 | 1.01 | 83.78 | 0.314 | 0.671 | 0.09 |
| Propionic acid | 0.86 ± 0.39 | 0.72 ± 0.35 | 2.26 | 83.75 | 0.027 | 0.080 | 0.20 |
| Succinic acid | 3.17 ± 1.14 | 2.65 ± 0.99 | 2.40 | 83.62 | 0.018 | 0.080 | 0.24 |
| Valeric acid | 0.07 ± 0.05 | 0.08 ± 0.07 | -0.77 | 83.45 | 0.445 | 0.671 | 0.07 |
|  | | | | | | | |
| **Clinical measures** | | | | | | | |
| LSAS-SR | 10.49 ± 10.47 | 74.63 ± 19.81 | -19.30 | 83.12 | < 0.001 |  | 2.01 |
| MADRS-S | 2.94 ± 3.66 | 13.08 ± 6.81 | -9.16 | 83.18 | < 0.001 |  | 0.92 |

**Table S3.** Intraclass correlation coefficients (ICCs) between the first and second baseline assessments in patients with social anxiety disorder (SAD) and healthy controls (HC).

|  | **Group** | **ICC** | ***F*** | **df1** | **df2** | ***P*** | **95% CI** | |
| --- | --- | --- | --- | --- | --- | --- | --- | --- |
|  |  |  |  |  |  |  | **Lower** | **Higher** |
| **Short-chain fatty acids** | | | | | | | | |
| Acetic acid | HC | 0.17 | 1.41 | 37 | 38 | 0.150 | -0.15 | 0.46 |
| Acetic acid | SAD | 0.43 | 2.52 | 42 | 43 | 0.002 | 0.16 | 0.65 |
| Butyric acid | HC | 0.47 | 2.75 | 37 | 38 | < 0.001 | 0.18 | 0.68 |
| Butyric acid | SAD | 0.45 | 2.61 | 42 | 43 | 0.001 | 0.17 | 0.66 |
| Caproic acid | HC | 0.47 | 2.79 | 36 | 37 | < 0.001 | 0.18 | 0.69 |
| Caproic acid | SAD | 0.58 | 3.77 | 42 | 43 | < 0.001 | 0.35 | 0.75 |
| Formic acid | HC | 0.56 | 3.56 | 37 | 38 | < 0.001 | 0.30 | 0.75 |
| Formic acid | SAD | 0.71 | 6.00 | 42 | 43 | < 0.001 | 0.53 | 0.83 |
| Isobutyric acid | HC | 0.39 | 2.27 | 37 | 38 | 0.007 | 0.09 | 0.63 |
| Isobutyric acid | SAD | 0.50 | 3.01 | 42 | 43 | < 0.001 | 0.24 | 0.69 |
| Isovaleric acid | HC | 0.34 | 2.05 | 37 | 38 | 0.015 | 0.03 | 0.60 |
| Isovaleric acid | SAD | 0.44 | 2.59 | 42 | 43 | 0.001 | 0.17 | 0.65 |
| Propionic acid | HC | 0.33 | 1.97 | 37 | 38 | 0.020 | 0.02 | 0.58 |
| Propionic acid | SAD | 0.33 | 1.98 | 42 | 43 | 0.014 | 0.04 | 0.57 |
| Succinic acid | HC | 0.60 | 4.05 | 37 | 38 | < 0.001 | 0.36 | 0.77 |
| Succinic acid | SAD | 0.69 | 5.51 | 42 | 43 | < 0.001 | 0.50 | 0.82 |
| Valeric acid | HC | 0.65 | 4.71 | 37 | 38 | < 0.001 | 0.42 | 0.80 |
| Valeric acid | SAD | 0.59 | 3.85 | 42 | 43 | < 0.001 | 0.35 | 0.75 |
|  | | | | | | | | |
| **Clinical measures** | | | | | | | | |
| LSAS-SR | HC | 0.89 | 17.67 | 40 | 41 | < 0.001 | 0.81 | 0.94 |
| LSAS-SR | SAD | 0.83 | 10.58 | 42 | 43 | < 0.001 | 0.70 | 0.90 |
| MADRS-S | HC | 0.70 | 5.63 | 40 | 41 | < 0.001 | 0.50 | 0.83 |
| MADRS-S | SAD | 0.71 | 5.91 | 42 | 43 | < 0.001 | 0.53 | 0.83 |

**Table S4.** Correlations between change in LSAS-SR scores and change in short-chain fatty acids.

| **Comparison** | **Short-chain fatty acids** | **Pearson r** | ***P*** | ***P_FDR_*** | ***n*** |
| --- | --- | --- | --- | --- | --- |
| **Pre- to post-treatment changes** | | | | | |
|  | Acetic acid | -0.19 | 0.23 | 0.68 | 43 |
|  | Butyric acid | 0.07 | 0.66 | 0.91 | 43 |
|  | Caproic acid | -0.04 | 0.79 | 0.91 | 43 |
|  | Formic acid | 0.11 | 0.49 | 0.88 | 43 |
|  | Isobutyric acid | 0.14 | 0.36 | 0.80 | 43 |
|  | Isovaleric acid | -0.04 | 0.81 | 0.91 | 43 |
|  | Propionic acid | 0.19 | 0.22 | 0.68 | 43 |
|  | Succinic acid | 0.35 | 0.02 | 0.20 | 43 |
|  | Valeric acid | 0.003 | 0.99 | 0.99 | 43 |
|  |  |  |  |  |  |
| **Post- to long-term treatment outcome changes** | | | | | |
|  | Acetic acid | -0.02 | 0.90 | 0.90 | 35 |
|  | Butyric acid | 0.18 | 0.29 | 0.90 | 35 |
|  | Caproic acid | 0.22 | 0.19 | 0.90 | 35 |
|  | Formic acid | -0.17 | 0.34 | 0.90 | 35 |
|  | Isobutyric acid | 0.10 | 0.56 | 0.90 | 35 |
|  | Isovaleric acid | 0.10 | 0.57 | 0.90 | 35 |
|  | Propionic acid | 0.03 | 0.89 | 0.90 | 35 |
|  | Succinic acid | -0.09 | 0.62 | 0.90 | 35 |
|  | Valeric acid | 0.07 | 0.70 | 0.90 | 35 |

**Table S5**. Insomnia Severity Index changes from pre- to post-treatment, and from post-treatment to long-term follow-up. Cohen’s *d* effect sizes are reported.

| **Comparison** | ***n*** | **Before** | **After** | ***t*** | **df** | ***P*** |  | **Cohen’s *d*** |
| --- | --- | --- | --- | --- | --- | --- | --- | --- |
| Pre- to post-treatment | 43 | 9.33 | 6.67 | 3.55 | 42 | 0.00096 |  | 0.54 |
| Post- to long-term follow-up | 38 | 6.29 | 6.45 | -0.23 | 37 | 0.82 |  | 0.04 |
